# Realizing high-capacity all-solid-state lithium-sulfur batteries using a low-density inorganic solid-state electrolyte

**DOI:** 10.1038/s41467-023-37564-z

**Published:** 2023-04-05

**Authors:** Daiwei Wang, Li-Ji Jhang, Rong Kou, Meng Liao, Shiyao Zheng, Heng Jiang, Pei Shi, Guo-Xing Li, Kui Meng, Donghai Wang

**Affiliations:** 1 Department of Mechanical Engineering, The Pennsylvania State University, University Park, PA 16802 USA; 2 Department of Chemical Engineering, The Pennsylvania State University, University Park, PA 16802 USA

**Keywords:** Batteries, Materials for energy and catalysis, Energy storage

## Abstract

Lithium-sulfur all-solid-state batteries using inorganic solid-state electrolytes are considered promising electrochemical energy storage technologies. However, developing positive electrodes with high sulfur content, adequate sulfur utilization, and high mass loading is challenging. Here, to address these concerns, we propose using a liquid-phase-synthesized Li_3_PS_4_-2LiBH_4_ glass-ceramic solid electrolyte with a low density (1.491 g cm^−3^), small primary particle size (~500 nm) and bulk ionic conductivity of 6.0 mS cm^−1^ at 25 °C for fabricating lithium-sulfur all-solid-state batteries. When tested in a Swagelok cell configuration with a Li-In negative electrode and a 60 wt% S positive electrode applying an average stack pressure of ~55 MPa, the all-solid-state battery delivered a high discharge capacity of about 1144.6 mAh g^−1^ at 167.5 mA g^−1^ and 60 °C. We further demonstrate that the use of the low-density solid electrolyte increases the electrolyte volume ratio in the cathode, reduces inactive bulky sulfur, and improves the content uniformity of the sulfur-based positive electrode, thus providing sufficient ion conduction pathways for battery performance improvement.

## Abstract (web summary)

Sulfur utilization in high-mass-loading positive electrodes is crucial for developing practical all-solid-state lithium-sulfur batteries. Here, authors propose a low-density inorganic solid-state electrolyte to improve the sulfur utilization in lab-scale Li-In||S all-solid-state cells.

## Introduction

Lithium-sulfur all-solid-state battery (Li-S ASSB) technology has attracted attention as a safe, high-specific-energy (theoretically 2600 Wh kg^−1^), durable, and low-cost power source for potential use in electric vehicles and drones^1,2^. Particularly, the ability of inorganic solid electrolytes (SEs) to prevent polysulfide dissolution endows Li-S ASSB with potential for achieving higher specific energy and a longer lifespan than conventional Li-S batteries using non-aqueous liquid electrolyte solutions^3,4^. To achieve high-specific-energy Li-S ASSBs beyond practical Li-ion batteries and Li-S batteries with liquid electrolytes, it is pivotal to realize high sulfur utilization >1000 mAh g^−1^ in sulfur cathode with high sulfur content >50 wt% (Supplementary Fig. 1)^5^. Various strategies have been proposed to fulfill this target, including improving cathode content uniformity and interfacial contact by developing tailored preparation approaches^6–9^, enhancing interfacial ionic transport by adding ionicliquids^10,11^, and increasing electronic/ionic transport by utilizing conversion-intercalation hybrid cathodes^12,13^ or SeS_x_ solid solution cathode^14^. However, whereas these strategies have improved sulfur utilization at low-sulfur-content conditions, few results realized high specific capacity in sulfur cathodes with a high sulfur content above 50 wt% (Supplementary Fig. 1).

**Fig. 1:**
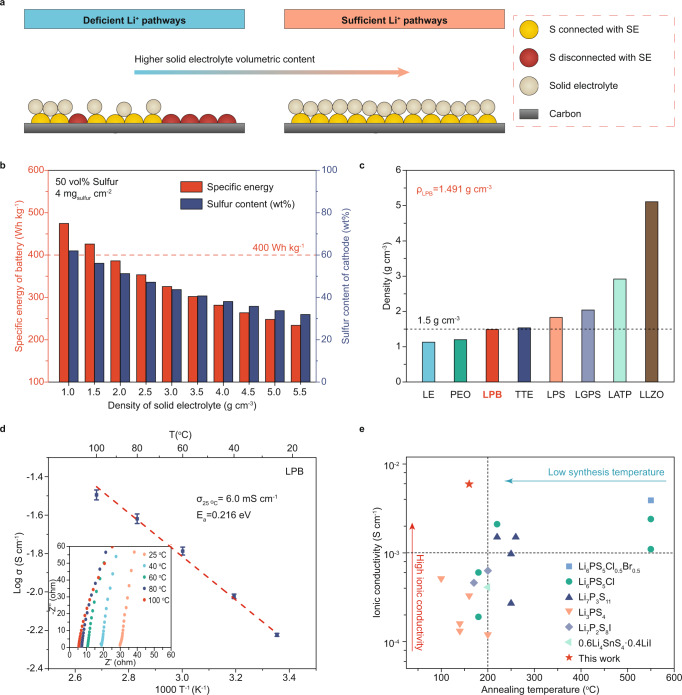
Physicochemical properties of the Li_3_PS_4_-2LiBH_4_ solid-state electrolyte. **a** Schematic illustration showing that the sufficiency of Li^+^ pathways increases upon increasing SE volume content (from left to right) in the sulfur cathode. **b** Dependence of specific energy at battery level and sulfur content at cathode level on the density of SE. The estimation was performed by assuming fixed volume ratios of cathode (or battery) components. The weight of the sulfur cathode, SE membrane, Li anode, and current collectors was considered for calculating the specific energy of Li-S ASSBs. **c** Comparison of density among LPB, non-aqueous liquid electrolyte solutions, polymer electrolytes, and inorganic SEs. LE: non-aqueous liquid electrolyte solutions; PEO: poly(ethylene oxide); LPB: Li_3_PS_4_-2LiBH_4_; TTE: 1,1,2,2-tetrafluoroethyl-2,2,3,3-tetrafluoropropyl ether; LPS: β-Li_3_PS_4_; LGPS: Li_10_GeP_2_S_12_; LATP: Li_1.3_Al_0.3_Ti_1.7_(PO_4_)_3_; LLZO: Li_7_La_3_Zr_2_O_12_. **d** Arrhenius plot for hot-pressed LPB SE pellet and the corresponding Nyquist impedance plot of the Al-C|LPB|Al-C coin cell tested from 25 to 100 ^o^C. The error bar represents the standard deviation of the measured ionic conductivity. **e** Comparison of ionic conductivity (20-30 °C) and synthesis temperature of LPB SE and reported liquid-phase-synthesized sulfide SEs^19–36^.

We deem the root cause of this challenge is the poor ionic transport within sulfur cathodes. Owing to the poor ionic conductivity of sulfur/Li_2_S, SE is the major component that regulates the Li^+^ transport in the cathode composed of sulfur, conductive carbon, and SE. Specifically, SE’s ionic conductivity governs the Li^+^ transport kinetics, while its volumetric content and particle size mainly determine the sufficiency of Li^+^ pathways (Fig. 1a and Supplementary Fig. 2). Especially at a high sulfur content of >50 wt%, the weight ratio of SE becomes as low as 35 wt%. Considering the high density of inorganic SE (typically >2 g cm^-3^), the volume ratio of SE drops even lower in the cathode (<35 vol%), giving rise to deficient Li^+^ pathways and hence mediocre sulfur utilization. Therefore, at high-sulfur-content conditions, utilizing low-density SE with small particle size can be a feasible approach to ensure high SE volume content (Supplementary Fig. 3), sufficient Li^+^ pathways, and thus high sulfur utilization to obtain high specific energy in Li-S ASSBs. Furthermore, to be competitive with commercial Li-ion batteries (~50 vol% active material loading in cathodes)^15,16^, it is imperative to employ SEs with a density <1.5 g cm^-3^ for fabricating >400 Wh kg^-1^ Li-S ASSBs with high sulfur weight ratio based on the cell design using thin Li and SE membranes (Fig. 1b, Supplementary Fig. 4 and Supplementary Note 1).

Herein, we demonstrate a strategy of using low-density, highly ionically conductive SE with small particle size to enable efficient ionic transport in high-sulfur-content cathodes and thus attain Li-S ASSBs with high specific capacity. As a proof of concept, the argyrodite glass-ceramic SE, Li_3_PS_4_-2LiBH_4_ (LPB), was synthesized via a liquid-phase method with a low measured density (1.491 g cm^-3^), high ionic conductivity (6.0 mS cm^-1^ at 25 °C), and small primary particle size (~500 nm). Consequently, the use of LPB SE enables high-performance Li-S ASSBs with a maximum discharge capacity of 1144.6 mAh g^−1^ at 167.5 mA g^-1^ using a positive electrode with a sulfur content of ~60 wt%. Meanwhile, stable cycling of a Li-S ASSB was also achieved with a high initial discharge capacity (1004.6 mAh g^−1^ at 837.5 mA g^-1^) and a low fading rate (~0.028% per cycle) for over 800 cycles with 77.5% capacity retention. Furthermore, through spectroscopic, microscopic, and electrochemical studies, we reveal that an excessively high sulfur volume ratio and meager SE volume ratio in sulfur cathodes would compromise cathode content uniformity, triggering the generation of inactive bulky sulfur particles, impeding ion transport and rendering low sulfur utilization.

## Results and discussion

### Physicochemical and electrochemical characterization of the Li_3_PS_4_-2LiBH_4_ solid-state electrolyte

The liquid-phase synthesis procedures of LPB SE are illustrated in Supplementary Fig. 5. Tetrahydrofuran (THF), a low-boiling-point solvent with low polarity, was selected as the reaction media to improve the ionic conductivity of sulfide SE products^17,18^. The obtained LPB SE powder possesses a low density of 1.491 g cm^-3^ at 21 °C, measured by the Helium Pycnometer. Intriguingly, the density of LPB is lower than other inorganic SEs and comparable to conventional non-aqueous liquid electrolyte solutions (Fig. 1c and Supplementary Table 1). Next, the ionic conductivity of LPB was measured at different temperatures from 25 to 100 °C by electrochemical impedance spectroscopy (EIS) using an Al-C|LPB|Al-C symmetric cell (Al-C denotes the carbon-coated aluminum foil). The LPB pellet prepared by cold-pressing delivered a high ionic conductivity of 3.8 mS cm^-1^ at 25 °C with a relative density of ~86.0% (Supplementary Fig. 6). Further enhancing the relative density of the prepared LPB pellet to ~91.5% (1.364 g cm^-3^, bulk density) through the hot-pressing process leads to a higher measured ionic conductivity of 6.0 mS cm^-1^ at 25 °C with a low activation energy of 0.216 eV (Fig. 1d). The result is among the highest ionic conductivities reported for liquid-phase-synthesized sulfide SEs (Fig. 1e and Supplementary Table 2)^19–36^. Notably, the synthesis temperature of LPB is 160 °C, lower than other liquid-phase-synthesized sulfide SEs with room-temperature ionic conductivity above 1 mS cm^-1^.

**Fig. 2:**
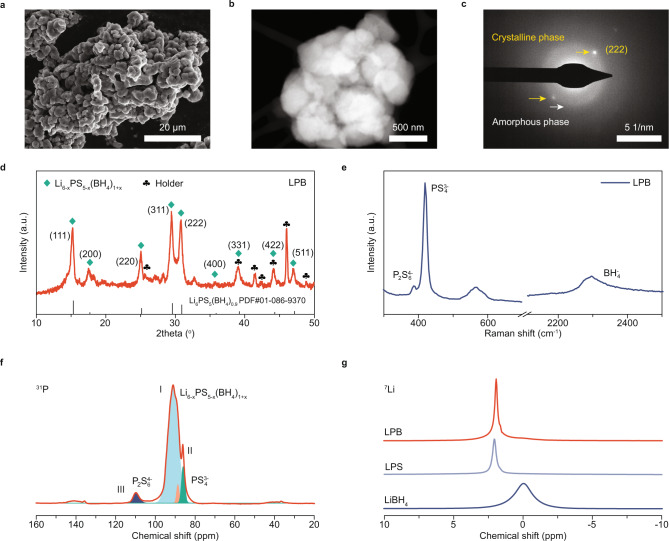
Morphological, structural, and compositional characterization of LPB. **a** SEM, **b** STEM, and **c** SAED images of LPB powders. **d** XRD patterns, **e** Raman spectra, and **f** ^31^P MAS NMR of LPB. **g** ^7^Li MAS NMR of LPB, LPS, and LiBH_4_. A Beryllium air-sensitive sample holder for XRD measurement was employed with its background patterns presented in Supplementary Fig. 11.

The morphology of LPB was investigated using scanning electron microscope (SEM) and transmission electron microscope (TEM). The SEM and energy-dispersive X-ray spectroscopy (EDS) mapping images show that the as-synthesized LPB powders are aggregates of ~5 μm small particles with uniformly distributed sulfur and phosphorous elements on the surface (Fig. 2a and Supplementary Fig. 7). Furthermore, scanning transmission electron microscope (STEM) and TEM images reveal that the 5-μm LPB particles are constituted of smaller nanoparticles of ~500 nm with no apparent porous structure (Fig. 2b and Supplementary Fig. 8) and Brunauer–Emmett–Teller (BET) surface area of ~3.705 m^2^ g^-1^ (Supplementary Fig. 9). The small particle size of as-synthesized LPB SE is favorable for attaining sufficient ionic percolation pathways in sulfur cathodes^15^. Moreover, both diffraction spots and diffused diffraction rings were observed in the selected area electron diffraction (SAED) pattern image (Fig. 2c, corresponding TEM image shown in Supplementary Fig. 10), suggesting LPB is composed of both crystalline and amorphous phases.

Next, we investigated the structure of the glass-ceramic LPB SE using the X-ray powder diffraction (XRD) technique and Raman spectroscopy. In Fig. 2d, diffraction peaks associated with the crystalline Li_6-x_PS_5-x_(BH_4_)_1+x_ (-1 < x ≤ 1) cubic argyrodite phase (Supplementary Note 2) were observed (Supplementary Fig. 11 illustrates the XRD patterns of the sample holder), agreeing with previously published results^37^. As reported in the literature^37^, while both crystalline and amorphous phases in LPB possess high ionic conductivity, the formation of the crystalline phase is beneficial for further enhancing LPB’s ionic conductivity. The calculated lattice parameter from the crystalline peaks is around 10.05 Å, and thus the theoretical density of the Li_6-x_PS_5-x_(BH_4_)_1+x_ crystal is ~1.59 g cm^-3^. Due to the amorphous phase’s presence, the LPB SE density shall be lower than the pure Li_6-x_PS_5-x_(BH_4_)_1+x_ crystal, explaining the measured lower density (1.491 g cm^-3^) of the as-obtained LPB powders. Meanwhile, low-intensity unknown peaks were also noticed in the XRD patterns, possibly originating from side reaction products or precursors/intermediate product residuals (Supplementary Fig. 12) during liquid-phase synthesis of the sulfide-based SEs^20,22^. Figure 2e presents the Raman spectra of the LPB SE. The high-intensity peak at ~418 cm^-1^ is mainly attributed to PS_4_^3-^ units in the glass-ceramic LPB, while the low-intensity peak at ~386 cm^-1^ stems from P_2_S_6_^4-^ units in the impurity, Li_4_P_2_S_6_. Besides, the broad peak at ~2300 cm^-1^ is ascribed to BH_4_^-^ units, similar to that in the high-temperature hexagonal phase of LiBH_4_ with weak electrostatic interaction between BH^-^ and Li^+^ ions^37,38^. This weak interaction is beneficial for improving Li^+^ mobility and achieving SEs with high ionic conductivity.

Solid-state nuclear magnetic resonance (NMR) spectroscopic characterization of LPB was further carried out to attain more details of the LPB’s local chemical structure and discern the impurities. As shown in the ^31^P NMR spectra (Fig. 2f), three distinct peaks, I, II, and III, were observed. The asymmetric high-intensity Peak I is associated with the phosphorous in Li_6-x_PS_5-x_(BH_4_)_1+x_ with a wide distribution of chemical shifts between 96 and 87 ppm. Peak I can be deconvoluted into two lines, implying the diverse chemical environment of PS_4_^3-^ tetrahedrons in the argyrodite Li_6-x_PS_5-x_(BH_4_)_1+x_ induced by the disorder in S^2-^/BH_4_^-^ sublattices. This disorder is favorable for improving the ionic conductivity in argyrodite SE, which might explain the good Li^+^ conductivity in LPB^39^. Peak II at ~86 ppm is attributed to the PS_4_^3-^ in β-Li_3_PS_4_ (LPS), generated from the heat treatment of residual Li_3_PS_4_-3THF^40^. Peak III at ~109 ppm originates from the P_2_S_6_^4-^ in Li_4_P_2_S_6_^41^, consistent with the Raman result above. Figure 2g presents the ^7^Li NMR spectra of LPB (~1.93 ppm) in comparison with LiBH_4_ (~0 ppm) and LPS (~2.07 ppm). In contrast to the broad peak at ~0 ppm in orthorhombic LiBH_4_, a sharp and narrow peak at ~1.93 ppm was observed in LPB, stemming from its significantly enhanced Li^+^ mobility.

### All-solid-state Li-S battery assembly and testing

**Fig. 3:**
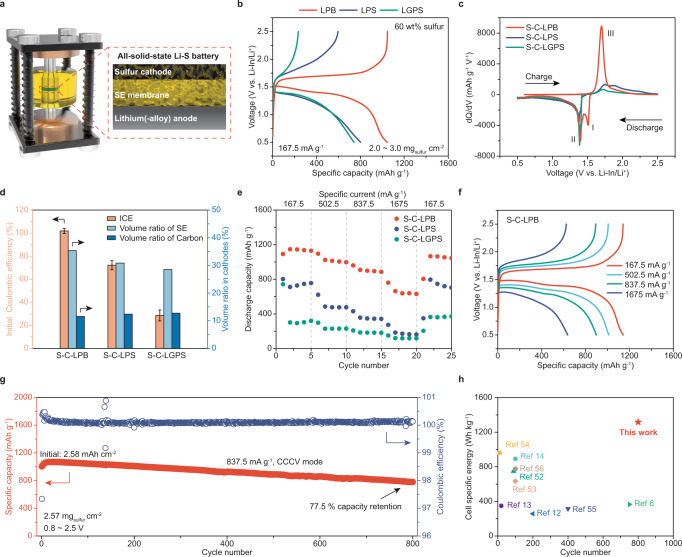
Battery testing of Li-In|LPB|S-C-SE all-solid-state cells. **a** Schematic illustration of the Swagelok cell and the configuration of Li-S ASSBs. **b** Galvanostatic discharge-charge curves of S-C-LPB, S-C-LPS, and S-C-LGPS cathodes upon 167.5 mA g^−1^ at 60 °C. **c** Differential capacity vs. voltage (dQ/dV) curves. **d** Comparison of initial Coulombic efficiency (ICE, charge capacity/discharge capacity) and volume ratios of SE and carbon in different cathodes. The error bar represents the standard deviation of average ICE. **e** Rate performance of different sulfur cathodes and **f** corresponding voltage profiles of S-C-LPB cathode at different rates. **g** Cycling performance of the Li-In|LPB|S-C-LPB cell at 837.5 mA g^−1^ under CCCV mode (cutoff current, 167.5 mA g^−1^; cutoff voltage, 2.5 V vs. Li-In/Li^+^) between 0.8 and 2.5 V at 60 °C. **h** Comparison of specific energy and cycle life of a cell with S-C-LPB cathode and literature-reported results^6,12–14,52–56^. The specific energy was estimated based on the weight of sulfur cathodes. Cell configurations, testing conditions, and performance of the selected literature-reported Li-S ASSBs are listed in Supplementary Table 3.

Solid-state sulfur cathodes and Li-S ASSBs were fabricated and evaluated. The cathode powders constituted of sulfur, conductive carbon (Ketjenblack EC-600JD, KB), and SEs were prepared by ball milling with a high sulfur content of ~60 wt% (S/KB/SE = 50/10/24, w/w/w). LPB, LPS, and Li_10_GeP_2_S_12_ (LGPS) SEs with particle sizes of ≤5 μm (Fig. 2a, b, and Supplementary Fig. 13) were used to fabricate the sulfur cathodes for comparison (S-C-LPB, S-C-LPS, and S-C-LGPS). The assembled Li-S ASSBs (Li-In|LPB|S-C-SE, assembled at 294 MPa) were tested in a Swagelok cell at 60 °C and 50–60 MPa under ambient air, with its configuration illustrated in Fig. 3a. Although metastable interphase can be formed between LPB pellet and Li metal to inhibit the degradation of LPB (Supplementary Figs. 14 and 15 and Supplementary Note 3), cell failure caused by lithium penetration may still occur under practical testing conditions^42–45^. Therefore, Li-In alloy (~0.62 V vs. Li/Li^+^) was employed as the anode due to its compatibility with inorganic SEs under high-areal-capacity and high-current-density conditions^46,47^.

Li-S ASSBs with an areal sulfur loading of 2.0 ~ 3.0 mg cm^-2^ were tested between 0.5 and 2.5 V. As shown in Fig. 3b, the S-C-LPB cathode delivered a higher discharge specific capacity (~1047.5 mAh g^−1^), higher initial Coulombic efficiency (ICE, charge capacity/discharge capacity, 100.2%) than S-C-LPS (~802.1 mAh g^−1^, 74.3%) and S-C-LGPS (~742.0 mAh g^−1^, 31.4%) cathodes at 167.5 mA g^-1^. Differential capacity curves are shown in Fig. 3c to elucidate the differences in the electrochemical process among all the cathodes. For the S-C-LPB cathode, prominent peaks attributed to redox reactions of sulfur/Li_2_S were observed at ~1.51 V (peak I, 2.13 V vs. Li/Li^+^) and ~1.40 V (peak II, 2.02 V vs. Li/Li^+^) during the discharging process and at ~1.70 V (peak III, 2.32 V vs. Li/Li^+^) during the charging process. In stark contrast, for the S-C-LPS and S-C-LGPS cathodes, peaks I and II become indistinguishable and shift to lower potentials with smaller areas, and peak III shifts to higher potentials, suggesting greater voltage polarization, lower sulfur utilization, and more sluggish reaction kinetics than S-C-LPB cathode. Such disparities in the cathode performance are attributed to the improved Li^+^ transport in S-C-LPB cathode in terms of transport kinetics determined by SE’s ionic conductivity and Li^+^ pathway sufficiency determined by SE’s volumetric content and particle size. Interestingly, we notice both ICE and initial discharge capacity, especially ICE, increase as the SE’s volumetric content ascends (Fig. 3d), while there is no apparent correlation between cathode performance and SE’s ionic conductivity/particle size (Figs. 1d, 2a, b and Supplementary Figs. 13 and 16). It might suggest that the low SE volume ratio induced Li^+^ pathway deficiency rather than ionic conductivity governed transport kinetics is the limiting factor that jeopardizes the performance of high-sulfur-content cathodes (i.e., 60 wt%).

The S-C-LPB cathode also shows better rate performance compared with S-C-LPS and S-C-LGPS cathodes (Fig. 3e), presenting higher discharge capacities of 1144.6, 1024.0, 907.8, and 663.0 mAh g^−1^ at 167.5, 502.5, 837.5 and 1675 mA g^-1^, respectively, with good reversibility under 60 °C (Fig. 3f). The improved performance of the S-C-LPB cathode at 167.5 mA g^-1^ is equivalent to a calculated Li-S cell specific energy of 1318.7 Wh kg^-1^ and volumetric energy density of 2561.8 Wh L^-1^ with an average discharge voltage of 1.935 V (vs. Li/Li^+^) (based on the weight/volume of sulfur cathodes, Supplementary Note 4). Since the redox reaction of LPB also contributed to capacity, we further investigated the solid electrolyte capacity contribution by cycling the Li-In|LPB|C-LPB cells (Super C/LPB = 30/70, w/w, denoted as C-LPB) following previously reported methods (Supplementary Fig. 17)^48–50^. The result shows that the high discharge capacity is mainly contributed by the lithiation of sulfur to Li_2_S (99.5% in the first cycle and >82% in the following cycles, Supplementary Note 5). In addition, the S-C-LPB cathode with a lower sulfur content of 50 wt% (S/KB/SE = 50/20/30, w/w/w) exhibited improved performance with >1300 mAh g^−1^ discharge capacity under 167.5 mAh g^−1^ at 60 °C, better than the cathodes using LPS and LGPS SEs (Supplementary Fig. 18). Good electrochemical performances of sulfur cathodes using LPS and LGPS can only be achieved when a sufficient amount of SEs is added (S/KB/SE = 50/10/40, w/w/w), giving rise to sufficient SE volume ratio and Li^+^ pathways (Supplementary Fig. 19). Note that the cathode composite powders were prepared using the conventional mechanical dry powder ball-milling method, and further performance improvement might be achievable by fine-tuning local interfacial contact among carbon, sulfur, and SE^51^.

The cycling stability of the S-C-LPB cathode was investigated as well. A Li-S ASSB cell with an areal sulfur loading of ~2.57 mg cm^-2^ was tested at 837.5 mA g^-1^ under a constant current, constant voltage (CCCV) mode (cutoff current, 167.5 mA g^-1^; cutoff voltage, 2.5 V) between 0.8-2.5 V (Fig. 3g). The S-C-LPB cathode cycled stably for over 800 cycles with a high initial discharge capacity of ~1004.6 mAh g^−1^ and a low fading rate of ~0.028% per cycle. The specific capacity gradually increased in the first 20 cycles to ~1068.1 mAh g^−1^, possibly attributed to the growth of capacity contribution from LPB’s redox reaction in the initial cycles (Supplementary Fig. 17). After 800 cycles, the discharge capacity remained at ~778.9 mAh g^−1^, corresponding to a discharge capacity retention of 77.5%. The cell performance is among the best in literature in terms of sulfur content, specific energy (based on the weight of sulfur cathodes), and cycling stability (Fig. 3h, Supplementary Fig. 1 and Supplementary Table 3)^6,12–14,52–56^. Upon further increasing sulfur mass loading to around 6 mg cm^-2^, the S-C-LPB cathode delivered a high initial discharge capacity of 999.7 mAh g^−1^ at 167.5 mA g^−1^ at 60 °C (Supplementary Fig. 20).

In addition to the electrochemical performance, we also investigated the capacity fading mechanism of the S-C-LPB cathode. As shown in the voltage profiles (Supplementary Fig. 21, corresponding to the cell in Fig. 3g), the voltage polarization of the cell during the charge/discharge narrows within the first few cycles, possibly due to the activation process associated with volume change-induced component redistribution. However, the polarization increases as cycling continues, leading to continuous capacity decay. Through EIS measurements and analysis, we found that the increment of the battery’s charge transfer resistance, which reached hundreds of ohms after over 1000 cycles (Supplementary Fig. 22 and Supplementary Table 4), is the culprit. Considering sulfide SE’s limited electrochemical stability voltage window^48,57,58^ and chemical reactivity against moisture^59^, we speculate that the primary cause of the growing voltage polarization and the ensuing capacity decay is the continuous electrochemical and chemical degradation of LPB SE during cycling. Thereafter, we investigated the redox activity of LPB and found that LPB would undergo relatively reversible lithiation/delithiation for over 100 cycles in the designated voltage window (i.e., 0.5-2.5 V vs. Li-In/Li^+^), explaining the improved cycle life of the S-C-LPB cathode (Supplementary Fig. 17 and 23 and Supplementary Note 6). Nevertheless, in the long-term cycling, the minor irreversible degradation may accumulate and lead to higher resistance, growth of voltage polarization, and capacity decay^57^. Moreover, the X-ray photoelectron spectroscopy (XPS) spectra of the cycled S-C-LPB electrode were further collected (Supplementary Fig. 24). While the observation of P-S_x_-P confirms the electrochemical oxidation of LPB, sulfate species in S 2*p* spectra suggest that the chemical degradation of LPB and active material might be another factor contributing to capacity decay. Supplementary Note 6 provides a more detailed analysis of capacity decay.

### Physicochemical and electrochemical characterizations of the sulfur-based positive electrodes

**Fig. 4:**
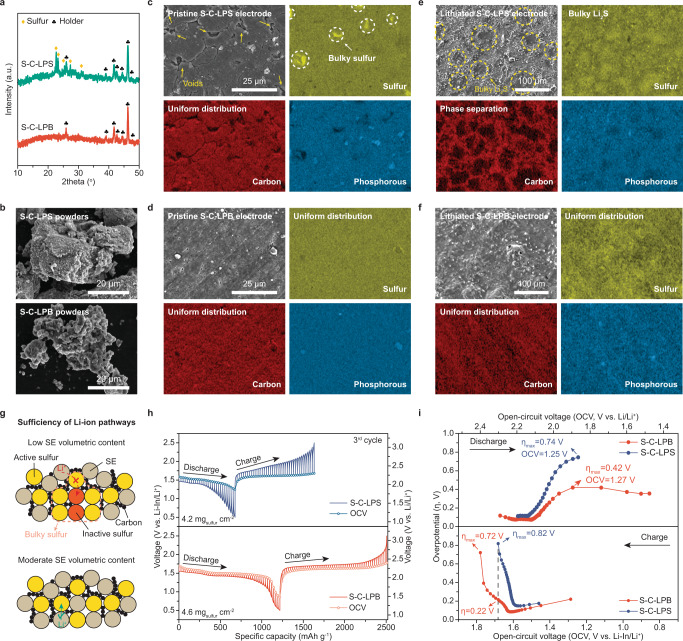
Physicochemical and electrochemical characterizations of the S-based positive electrode. **a** XRD patterns of S-C-LPS and S-C-LPB cathode powders. **b** SEM images of S-C-LPS and S-C-LPB cathode powders. **c**–**f** Ex-situ SEM and EDS mapping images of **c** pristine S-C-LPS cathode, **d** pristine S-C-LPB cathode, **e** lithiated S-C-LPS cathode, and **f** lithiated S-C-LPB cathode. **g** Schematic illustration of the influence of SE’s volumetric content on interparticle Li^+^ transport and formation of inactive bulky sulfur particles. **h** GITT and OCV curves of S-C-LPB and S-C-LPS cathodes at the third cycle. Current pulses of 50.25 mA g^−1^ for 30 mins were employed, followed by 4 h resting. **i** Overpotential profiles of S-C-LPB and S-C-LPS cathodes from the GITT measurement.

To clarify the underlying reasons behind the improved electrochemical performance of the S-C-LPB cathode, we first performed XRD and SEM analyses of S-C-LPB and S-C-LPS at both material and electrode levels. The XRD patterns shown in Fig. 4a indicate the presence of crystalline sulfur particles in S-C-LPS cathode powders, in stark contrast to the amorphous sulfur particles in the S-C-LPB cathode. Moreover, it is found that the morphology and content uniformity of sulfur cathodes are correlated with the volumetric contents of SE and sulfur. The volumetric ratios of different cathode components, i.e., sulfur, carbon, and SE, are calculated and illustrated in Supplementary Fig. 25. A higher SE volume ratio and lower sulfur volume ratio in S-C-LPB are obtained compared with S-C-LPS at the same weight contents, thanks to the lower density of LPB. As shown in the SEM images (Fig. 4b), the S-C-LPB cathode aggregate powders are smaller than the S-C-LPS aggregate. Upon pressing the powders into electrodes, the formed S-C-LPB cathode made from small powders seems relatively denser than the S-C-LPS sulfur cathode with voids on the surface (Fig. 4c, d). In addition, distinct from the homogeneously distributed sulfur signals on the pristine S-C-LPB electrode surface, locally high-intensity sulfur signals are detected (marked in dashed circles) in the EDS mapping of the S-C-LPS electrode, indicating the presence of bulky sulfur particles. The absence of the micrometer bulky sulfur particles might help reduce the aggregate size of S-C-LPB powders (Supplementary Fig. 26). After lithiation, massive aggregated Li_2_S emerges and separates from carbon (Fig. 4e) in the S-C-LPS cathode. Given the poor electronic and ionic conductivity of sulfur/Li_2_S, the lithiation/delithiation of bulky sulfur/Li_2_S is kinetically less favorable than small sulfur/Li_2_S particles, thus leading to higher resistance and lower sulfur utilization of the S-C-LPS cathode. In stark contrast, the S-C-LPB cathode exhibits good content uniformity with homogenous distribution of all elements after lithiation (Fig. 4f). Similarly, upon further decreasing LPB’s volume ratio to 27.9 vol% and increasing sulfur volume ratio to 59.2 vol% in S-C-LPB (65 wt% of sulfur, denoted as S-C-LPB-65), we also observed larger powder size, worsened content uniformity, and deteriorated battery performance with lower discharge capacity (678 mAh g^−1^) and poor ICE (62.3%) in the obtained S-C-LPB-65 cathode (Supplementary Figs. 27, 28 and 29). It demonstrates that this phenomenon is independent of the type of SE but related to the SE volume ratio. Therefore, these results collectively reveal that an adequate volume ratio of SE is the prerequisite for avoiding inactive bulky sulfur particles, achieving good content uniformity, constructing sufficient ionic transport pathways, and thus attaining high-performance sulfur cathodes (Fig. 4g and Supplementary Fig. 30). Note that, in addition to SE volume ratio, the large particle size of SE may likewise cause deficient Li^+^ transport pathways^15^ and thus the mediocre performance of sulfur cathodes by inducing poor cathode content uniformity with inactive bulky sulfur particles and phase separation between carbon and large SE powders (Supplementary Fig. 31, 32 and 33 and Supplementary Note 7).

Furthermore, the galvanostatic intermittent titration technique (GITT) was performed using Li-S ASSBs with S-C-LPB and S-C-LPS cathodes to explicate sulfur cathodes’ electrochemical behaviors during charge/discharge and identify their correlations with the cathode features observed above. Figure 4h depicts the obtained GITT and open-circuit voltage (OCV, defined as the voltage reached after each 4-h resting) profiles (Supplementary Fig. 34a). Two voltage plateaus at ~1.59 and ~1.48 V (~2.21 and ~2.10 V vs. Li/Li^+^) during lithiation, which might involve the reduction of sulfur to lithium polysulfide (first plateau) and then to Li_2_S (second plateau), were noticed in both samples, agreeing with the differential capacity curves (Fig. 3c). Note that the two reduction plateaus observed in the S-C-LPB cathode were also found in S-C-LPS and S-C-LGPS cathodes with a lower sulfur content of 50 wt% and higher SE content of 40 wt% (Supplementary Fig. 19a, b). Next, assuming that the batteries reached thermodynamic equilibrium after each 4-h resting and the electrochemical reactions in both cathodes are identical at the same OCV, we can compare the amount of electrochemically active sulfur in S-C-LPB and S-C-LPS cathodes. To do so, we calculated the discharge/charge specific capacity of cells with both cathodes within certain OCV ranges that are 1.57 ~ 1.28 V (2.19 ~ 1.9 V vs. Li/Li^+^) during lithiation and 1.58 ~ 1.68 V (2.20 ~ 2.30 V vs. Li/Li^+^) during delithiation (Supplementary Fig. 34b, c). The higher lithiation/delithiation specific capacity of the S-C-LPB cathode (860/950 mAh g^−1^) than the S-C-LPS cathode (604/806 mAh g^−1^) implies more electrochemically active sulfur in the S-C-LPB cathode (Supplementary Fig. 34d and Supplementary Note 8). In addition, significantly lower overpotential and IR drop are observed in the cell with the S-C-LPB cathode during lithiation/delithiation (Fig. 4i and Supplementary Fig. 34e, f), suggesting lower reaction resistance and improved Li^+^ transport kinetics in the S-C-LPB cathode than in the S-C-LPS cathode. Together, the above analysis demonstrates that sufficient Li^+^ transport pathways and fast Li^+^ transport kinetics are achieved in the S-C-LPB cathode.

In summary, we have demonstrated a strategy of employing SE with low density and high ionic conductivity for achieving high specific capacity in Li-S ASSBs. A low-density LPB SE with a high room-temperature ionic conductivity was prepared by a liquid phase method. Distinct from conventional inorganic SEs (i.e., LPS and LGPS) with high density, the low density of LPB SE enables sulfur cathodes with a higher SE volume ratio, diminished bulky sulfur particles, and good content uniformity; meanwhile, the high ionic conductivity of LPB SE endows the sulfur cathode with low resistance and good ionic transport kinetics. In this manner, efficient ionic transport can be established to achieve good sulfur utilization in sulfur cathodes with high sulfur content and high mass loading. Consequently, high-sulfur-content, high-areal-mass-loading sulfur cathodes with good sulfur utilization can be achieved. The high-performance sulfur cathodes enabled by the LPB SE can potentially allow Li-S ASSB to reach a high specific energy above 300 Wh kg^-1^ if combined with thin Li and SE membranes.

## Methods

### Synthesis of solid electrolytes

THF (anhydrous, ≥99.9%, Sigma-Aldrich), Li_2_S (99.98%, Sigma-Aldrich), P_2_S_5_ (99%, Sigma-Aldrich), LiBH_4_ (95%, Sigma-Aldrich), and LiBH_4_ solution (2.0 M in THF, Sigma-Aldrich) were purchased and used without any further treatment. Experiments were performed in an argon-filled glovebox (H_2_O <1 ppm, O_2_ <5 ppm). To prepare the LPB SE, Li_2_S and P_2_S_5_ with a molar ratio of 3 to 1 were added to anhydrous THF and stirred for 24 h at 25 °C. Next, the LiBH_4_ solution with the desired amount (LiBH_4_:Li_2_S:P_2_S_5_ = 4:3:1, molar ratios) was added to the above suspension using a single-channel high-precision pipette. After stirring for 48 h, the obtained suspension was transferred to a Schlenk flask and dried under vacuum for 2 h at 100 °C to remove the solvent. Finally, the powders were collected and annealed at 160 °C for 3 h in the argon atmosphere to obtain the LPB glass-ceramic argyrodite SE. LPS (β-Li_3_PS_4_) was prepared via the liquid-phase method following the procedures in the literature^19^. Typically, Li_2_S and P_2_S_5_ with a stoichiometry of 3 to 1 were added to anhydrous THF and stirred for 24 h at 25 °C. Next, the precipitation product was collected by centrifuge and washed with anhydrous THF for three times. Finally, the collected white precipitation was dried at 140 °C under vacuum for 3 h, obtaining LPS powders. In addition, Li_10_GeP_2_S_12_ (LGPS, >99.9%) and Li_6_PS_5_Cl (LPSC, >99.9%) were purchased from MSE Supplies LLC.

### Ionic conductivity measurement

Al-C|SE|Al-C symmetric cells were assembled to measure the ionic conductivity of SEs via analysis of the EIS measurements. In detail, ~150 mg LPB powders were sandwiched between two Al-C foils and pressed at ~500 MPa in a stainless-die sleeve at 25 °C for 10 mins. Next, it was heated to 160 °C and pressed again at ~500 MPa for another 20 mins, obtaining the hot-pressed LPB pellet. LPS and LGPS pellets were prepared similarly without being heated and pressed at high temperatures. Finally, the fabricated Al-C|SE|Al-C pellet was loaded into a CR2032 coin cell, and EIS measurement of the cell was performed from 0.1 Hz to 1 MHz with a bias of 10 mV under the potentiostatic mode in the logarithmic manner (10 points per decade) on a Solartron Modulab at 25, 40, 60, 80, and 100 °C.The cell was rested for around 30 mins at the testing temperature in the climatic chamber (Tenney) before each EIS measurement.

### Materials characterization

Helium Pycnometry was used to measure the density of LPB. Specifically, ~0.4 g of LPB SE powders were loaded and sealed into the sample cup in an argon-filled glovebox (H_2_O <1 ppm, O_2_ <5 ppm) and then transferred to the Helium Pycnometry instrument with air exposure time <1 min. The measurement was carried out at ~21 °C for ten times. SEM and EDS mapping images were collected on a Thermo Scientific Apreo SEM instrument and an ESEM Q250 instrument. The samples were exposed to air for <5 min during sample transfer. TEM and STEM images and SAED patterns of the LPB powders were collected on an FEI Talos F200X TEM with an accelerating voltage of 200 kV. The TEM samples were prepared by loading dry LPB powders onto the Cu grids in an argon-filled glovebox (H_2_O <1 ppm, O_2_ <5 ppm). The grids were then transferred to a TEM holder and inserted into the microscope with <1 min air-exposure time. The BET surface area of LPB was measured by a Micrometrics ASAP 2020 physisorption analyzer. A Rigaku Miniflex II spectrometer with Cu Kα radiation was used to collect the XRD patterns of the samples. An XRD air-sensitive sample holder with a Beryllium window (Rigaku Corp.) was used to load the sample powders in an argon-filled glovebox (H_2_O <1 ppm, O_2_ <5 ppm). Raman spectra of LPB powders were collected on a Horiba LabRam HR Evolution Vis-NIR optimized & AIST-NT Scanning Probe using the 532 nm laser. To prevent LPB from reacting with moisture in the air, we sealed the powdered sample between two transparent glass slides under argon. The XPS spectra and depth profiling characterization were carried out on a PHI VersaProbe II Scanning XPS Microprobe. We employed a vacuum transfer vessel to transfer the samples from the argon-filled glovebox (H_2_O <1 ppm, O_2_ <5 ppm) to the XPS instrument. Additionally, ^7^Li and ^31^P MAS NMR spectra were characterized on an Avance-III-HD ss500 instrument with a 4-mm Bruker CPMAS probe at a spinning speed of 10 kHz. CaHPO_4_·2H_2_O and LiCl were used as references for ^31^P and ^7^Li NMR spectra, respectively.

### Cathode preparation

Carbon-sulfur composite was prepared by mixing sulfur (≥99.0%, Sigma-Aldrich) and KB (Ketjenblack EC-600JD, AkzoNobel) with desired weight ratios (S:KB = 50:10 or 50:20, w/w) and heated under 160 °C for 10 h. The as-obtained carbon-sulfur composite powders (83.3 wt% or 71.4 wt% of sulfur) and SEs (LPB, LPS, or LGPS) with desired weight ratios were then mixed by mechanical ball milling under 350 rpm for 10 h in 45 ml ZrO_2_ jars (FRITSCH PULVERISETTE. 7 premium line), obtaining sulfur cathode powders. The C-SE cathode powders were prepared similarly by mixing 70 wt% of SE (i.e., LPB or LPSC) and 30 wt% Super C using mechanical ball milling under 350 rpm for 10 h in 45 ml ZrO_2_ jars. All process was performed under an inert atmosphere filled with Argon (H_2_O <1 ppm, O_2_ <5 ppm).

### All-solid-state cell fabrication and electrochemical testing

ASSBs were assembled in Swagelok cells with two stainless-steel rods as current collectors inside an Argon-filled glovebox (H_2_O <1 ppm, O_2_ <5 ppm). In detail, 80 mg of SE powders were firstly pressed at 100 MPa for 1 min in the cell to form a pellet. Next, cathode powders with the desired amount were uniformly spread onto the pellet’s surface and pressed at 294 MPa for another 3 mins. The average thickness of the SE pellet is ~600 μm. Lithium foil (3 ~ 4 mg; cut from Li chips, 99.9%, 0.6 mm thick, China Energy Lithium Co., Ltd) and indium foil (10 mm in diameter, 0.127 mm thick, 99.99%, Sigma-Aldrich) were then successively pressed together and attached to the other side of the pellet at 100 MPa for 1 min. Finally, the cells were compressed by three insulated bolts at 50 ~ 60 MPa, removed from the glovebox, and tested using a Landt cycler at 60 °C under ambient air in a forced air oven (Across International). The applied specific current and the measured specific capacity are based on the mass of sulfur in the positive electrode. Note that the Swagelok cell could not fully prevent the electrode from contacting ambient air. The EIS spectra were obtained on a Solartron Modulab from 0.1 Hz to 1 MHz under the potentiostatic mode in the logarithmic manner (10 points per decade) with a bias of 5 mV at 60 °C. Before carrying out the EIS measurement, the cell was rested at 60 °C for 1 h. The linear sweep voltammetry of Li-In|SE|C-SE cells was carried out on a Solartron Modulab with a scan rate of 0.05 mV s^-1^ from open-circuit voltage to 4.38 or -0.62 V vs. Li-In/Li^+^ (5 or 0 V vs. Li/Li^+^) at 60 °C. For GITT measurements, the cells were firstly cycled at 167.5 mA g^-1^ for two cycles between 0.5 ~ 2.5 V and then tested with a pulse current of 50.25 mA g^-1^ for 30 mins and a resting time of 4 h at 60 °C. All electrochemical tests have been carried out at least two times and most of them have been tested for three times.

### Lithium symmetric cell fabrication and testing

Li|LPB|Li symmetric cell was fabricated inside an argon-filled glovebox (H_2_O <1 ppm, O_2_ <5 ppm). Hot-pressed LPB pellets (~1.3 mm thick) were prepared following the procedure described in the ionic conductivity measurement section. Two pieces of 100-μm thick lithium chips (99.9%, China Energy Lithium Co., Ltd) with a diameter of 10 mm on Cu foils (≥99.8%, 9 μm thick, MTI corporation) were attached to both sides of the SE pellet by pressing at 100 MPa for 2 mins. The symmetric cell was then loaded into a 10-mm diameter pressure-controlled split coin cell (MTI corporation) for electrochemical testing. A mechanical jig fixture was used to control the pressure of the cell to 6 ~ 8 MPa. Finally, the cell was tested using a Landt cycler under different current densities of 0.1, 0.25, 0.5, 0.75, and 1.0 mA cm^-2^ at 25 °C with the duration of each discharge/charge process fixed at 1 h. Three symmetric cells were assembled for testing.

## Supplementary information

Supplementary Information

## Data Availability

The datasets generated during and/or analysed during the current study are available from the corresponding author on reasonable request.
