# Supplementary material for: Realizing high-capacity all-solid-state lithium-sulfur batteries using a low-density inorganic solid-state electrolyte
Source: Nat Commun. 2023 Apr 5;14:1895. doi: 10.1038/s41467-023-37564-z (PMC10076334; doi:10.1038/s41467-023-37564-z)
Supplement: Supplementary file 1 — Supplementary Information [file 41467_2023_37564_MOESM1_ESM.pdf]

Supplementary information

Realizing high-capacity all-solid-state lithium-sulfur batteries using a low-density inorganic solid-state electrolyte

Daiwei Wang,[1] Li-Ji Jhang,[2] Rong Kou,[1] Meng Liao,[1] Shiyao Zheng,[1] Heng Jiang,[1] Pei Shi,[2] Guo-Xing Li,[1] Kui Meng,[1] and Donghai Wang[1]*

[1]Department of Mechanical Engineering, The Pennsylvania State University, University Park, PA 16802, USA. [2]Department of Chemical Engineering, The Pennsylvania State University, University Park, PA 16802, USA. *Corresponding author. Email: dwang@psu.edu

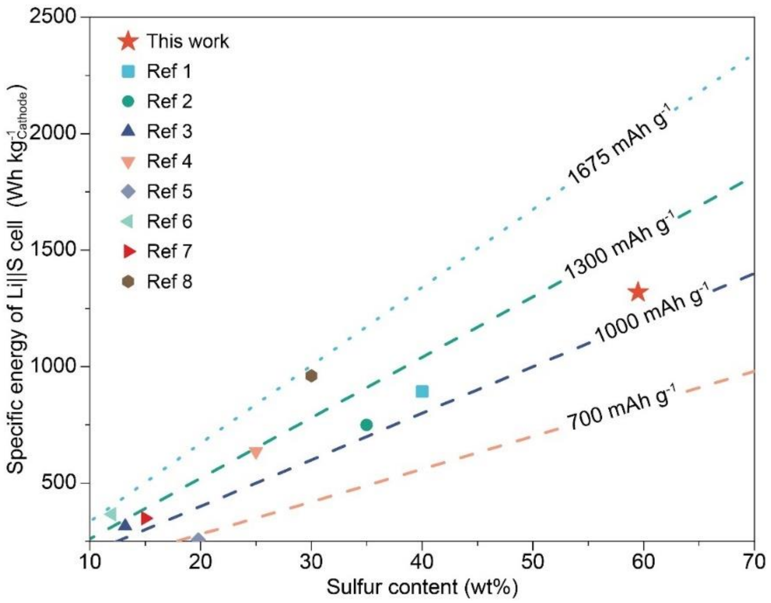

Supplementary Figure 1. Illustration of the influence of sulfur content and sulfur utilization on the specific energy (based on the cathode weight) of lithium-sulfur all-solid-state batteries (Li-S ASSBs). Cell configurations, testing conditions, and performance of the selected literature-reported Li-S ASSBs are listed in Supplementary Table 3.

The estimation of the specific energy is based on the following equation:

$$\text{Specific energy} = \frac{m\ \text{mg cm}^{-2} \times w\% \times a\ \text{mAh g}^{-1} \times U\ \text{V}}{m\ \text{mg cm}^{-2}} \times \frac{1\ \text{Ah}}{1000\ \text{mAh}} \times \frac{1000\ \text{g}}{1\ \text{kg}} = Uaw\%\ \text{Wh kg}^{-1}$$

where m is the cathode mass loading (mg cm^{-2}), w wt% is the gravimetric sulfur content in the cathode, a is the specific capacity (mAh g^{-1}), and U is the average voltage of the Li||S cell. We assumed the average voltage (U V) is 2 V, and the specific capacity (a mAh g^{-1}) and sulfur content (w wt%) of the literature reported Li-S ASSBs used for calculation are listed in Supplementary Table 1.

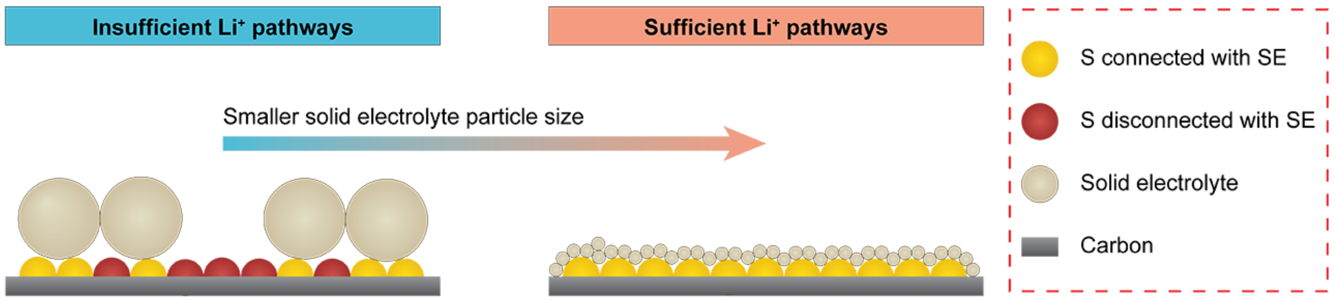

Supplementary Figure 2. Schematic illustration of the influences of SE's particle size ion Li^+ transport and thus sulfur utilization.

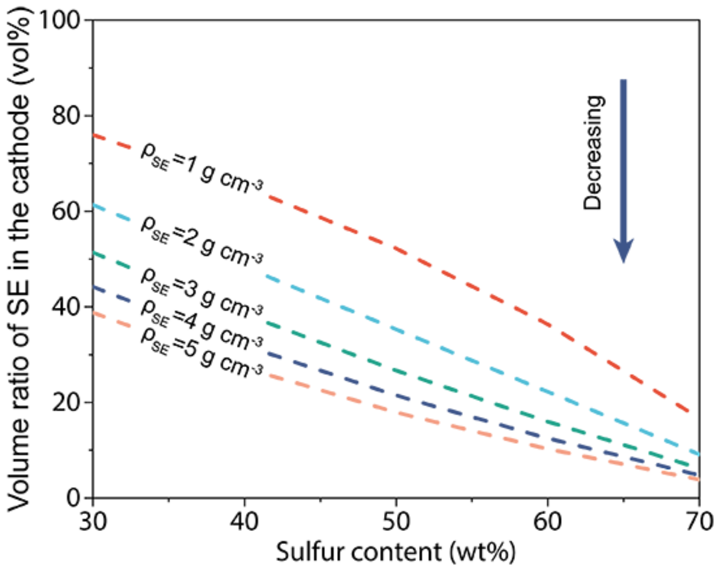

Supplementary Figure 3. Illustration of the relationship between sulfur's gravimetric content (wt%) and SE's volumetric content (vol%) under different solid electrolyte (SE) densities.

To illustrate how sulfur's weight ratio and SE's density affect SE's volumetric content in the cathode, we assume that the weight ratio of carbon and sulfur are 15 wt% and x wt%. Moreover, the density of carbon, sulfur, and SE are 1.9, 2.07, and ρ g cm^{-3}, respectively. Hence, the volume ratio of SE could be calculated according to the following equation:

$$\text{SE content (vol\%)}=\frac{\frac{(100\text{-}15\text{-}x)}{\rho}}{\frac{(100\text{-}15\text{-}x)}{\rho}+\frac{15}{1.9}+\frac{x}{2.07}}$$

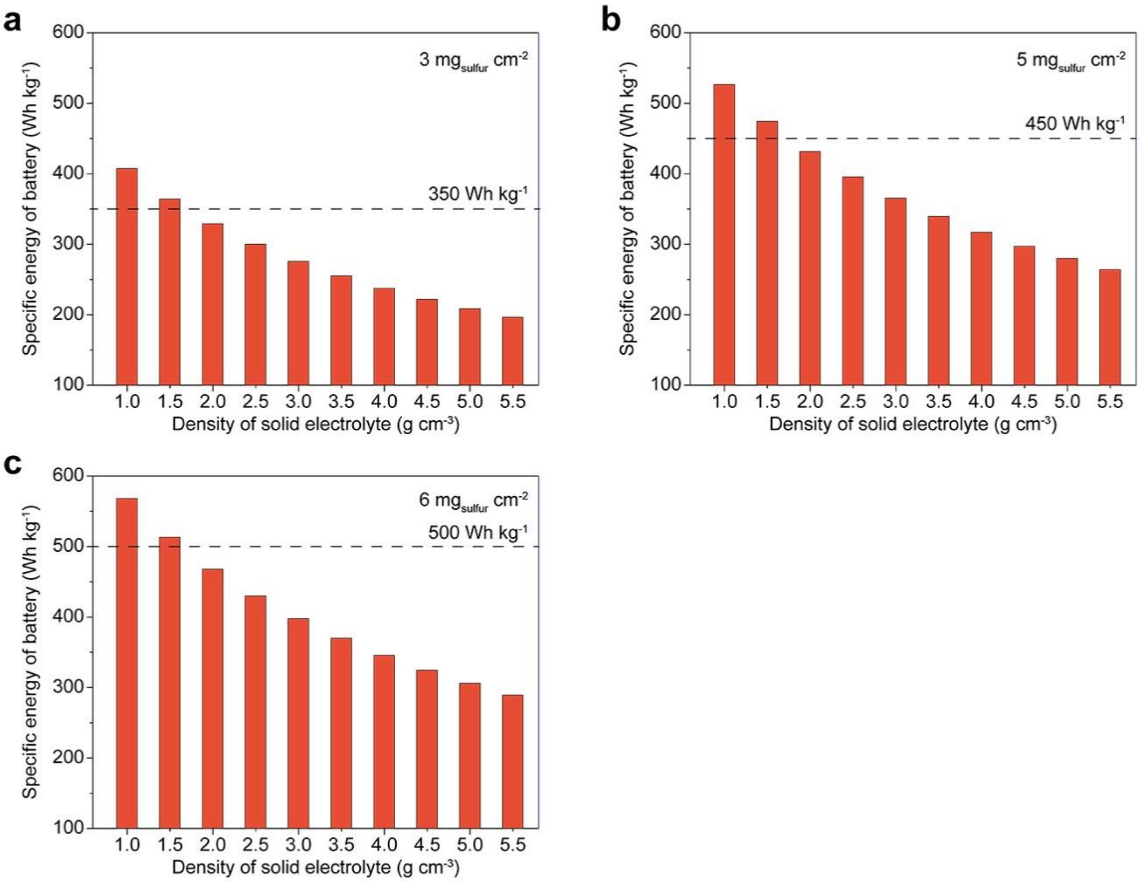

Supplementary Figure 4. Illustration of the influence of SE's density on the cell-level specific energy of Li-S ASSBs (based on the weight of cathode, anode, SE membrane, and current collectors) using sulfur cathodes (50 vol% of sulfur) with areal sulfur loading of (**a**) 3, (**b**) 5, and (**c**) 6 mg$_{sulfur}$ cm^{-2}. Calculation details are provided in Supplementary Note 1.

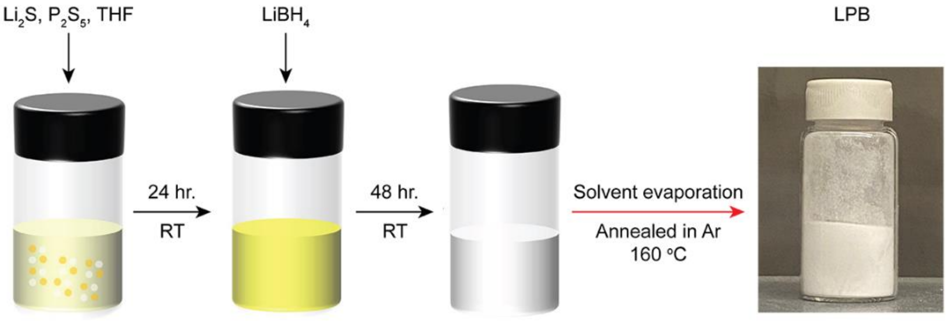

Supplementary Figure 5. Schematic illustration of the liquid-phase synthesis of Li_3PS_4-2$LiBH_4$ (LPB) SE.

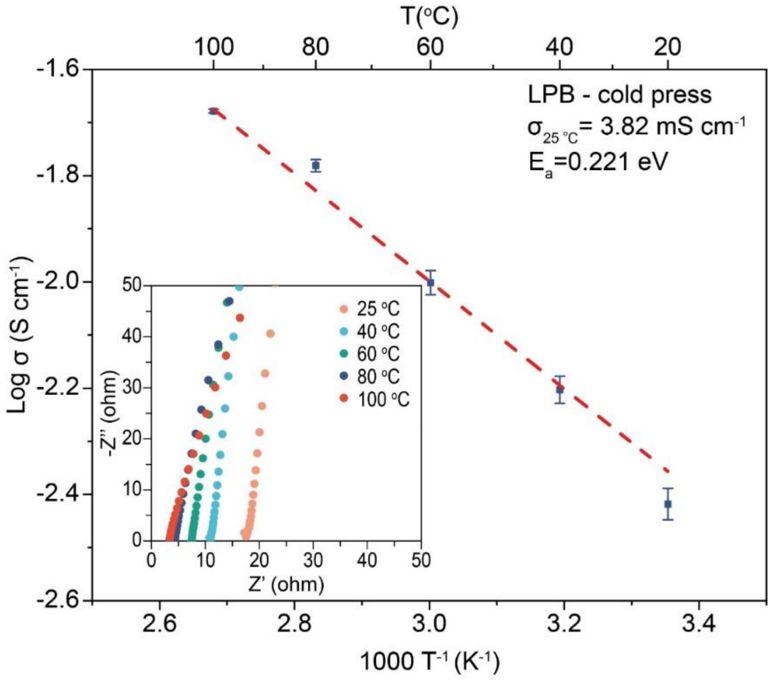

Supplementary Figure 6. Arrhenius plot for cold-pressed LPB SE pellet and the corresponding Nyquist impedance plot of the Al-C|LPB|Al-C (Al-C stands for carbon-coated aluminum foil current collector) coin cell tested at different temperatures (i.e., 25, 40, 60, 80, 100 ^{o}C). The error bar represents the standard deviation of the measured ionic conductivity.

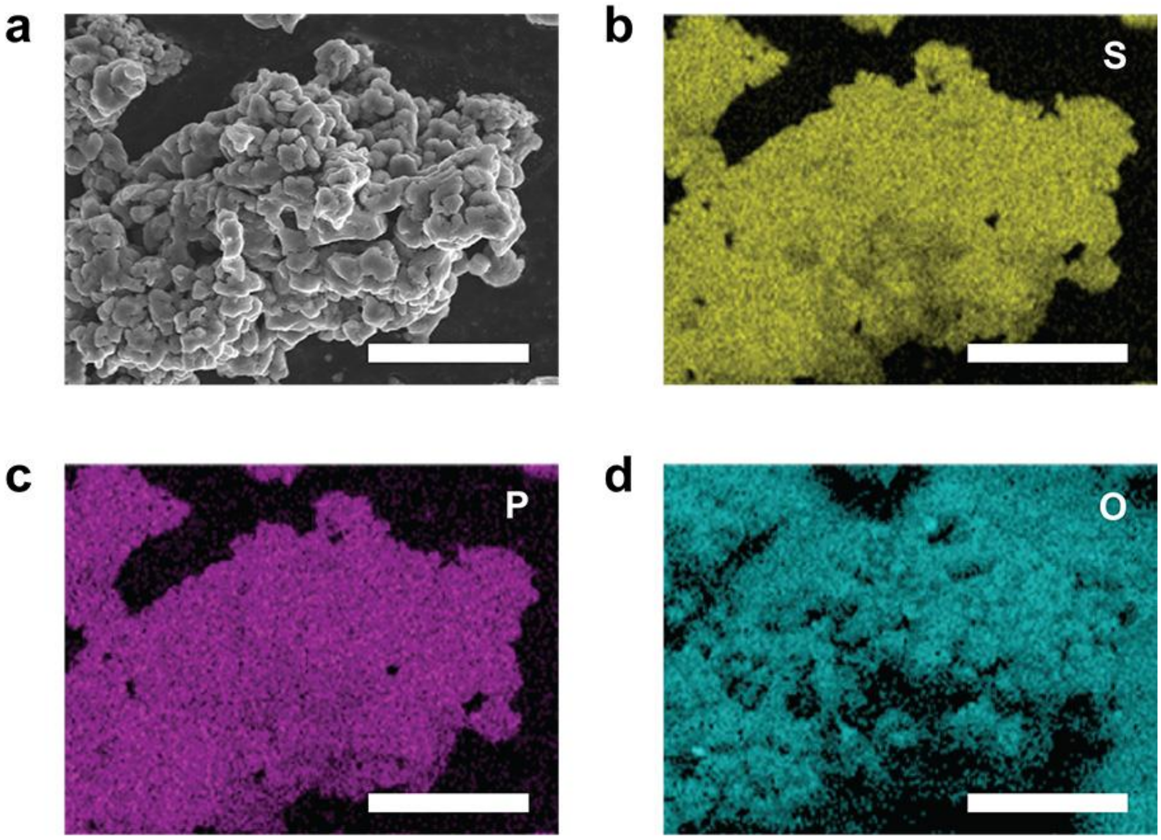

Supplementary Figure 7. (**a**) Scanning electron microscopy (SEM) and (**b-d**) energy-dispersive X-ray spectroscopy (EDS) mapping images of LPB powders. The scale bars are 20 μm.

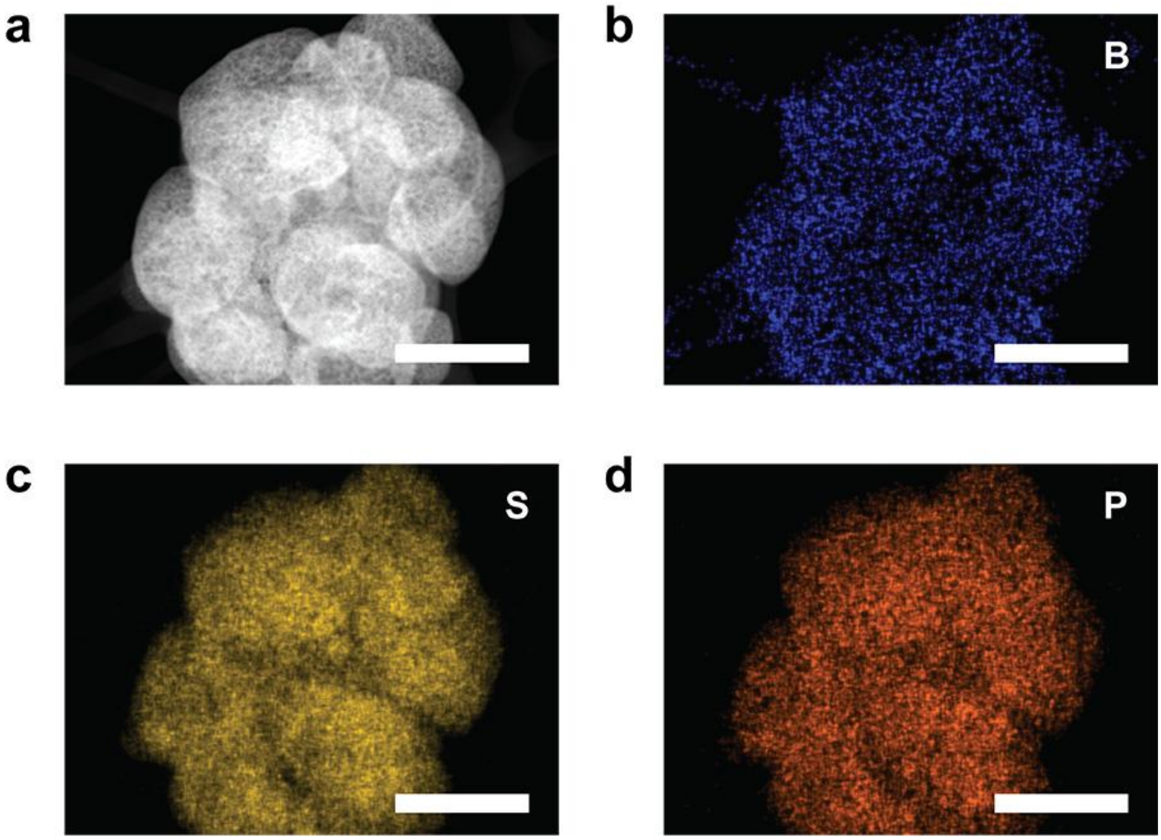

Supplementary Figure 8. Scanning transmission electron microscopy (STEM) and EDS mapping of LPB powders. The scale bars are 500 nm.

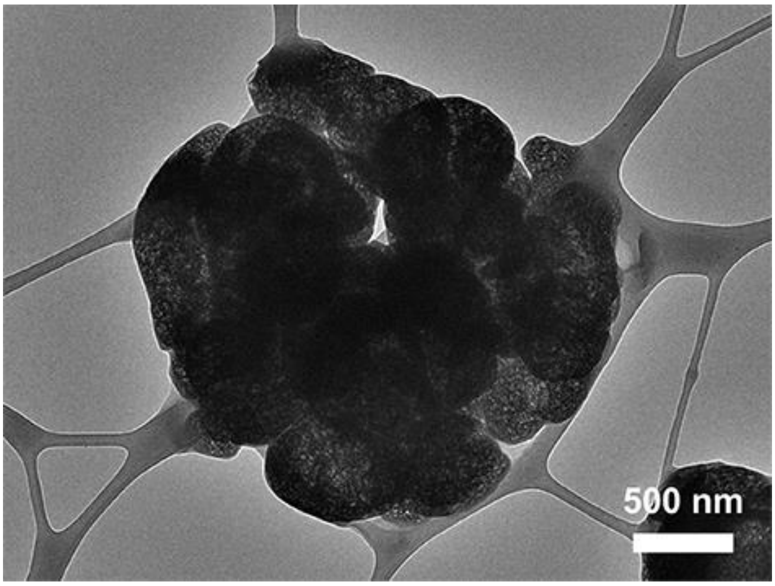

Supplementary Figure 9. Transmission electron microscopy (TEM) images of LPB powders corresponding to the selected area diffraction pattern in Figure 2c. The porous structure observed in this image is caused by electron beam damage.

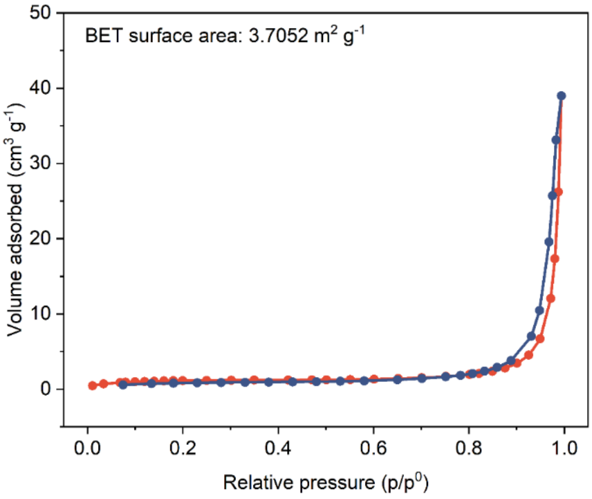

Supplementary Figure 10. N_2 adsorption/desorption isotherms of LPB powders at 77 K.

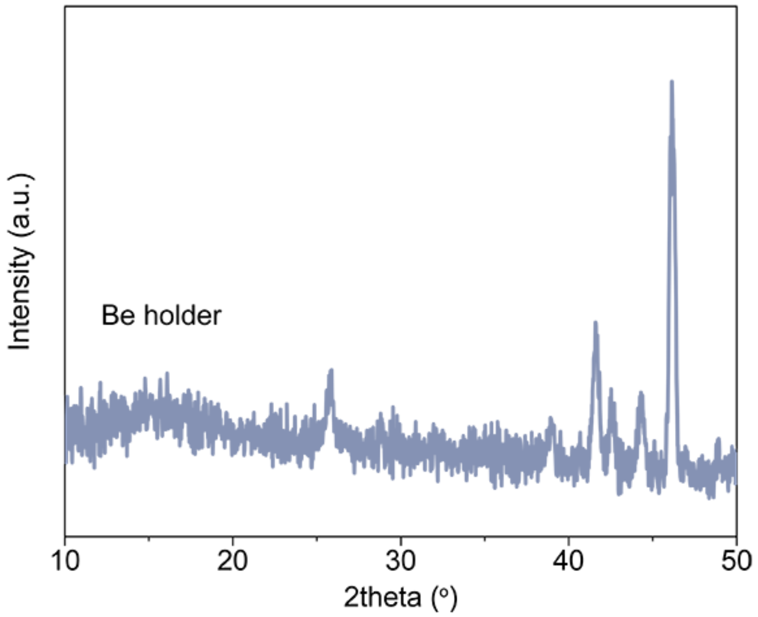

Supplementary Figure 11. X-ray powder diffraction (XRD) pattern of the Beryllium air-sensitive sample holder.

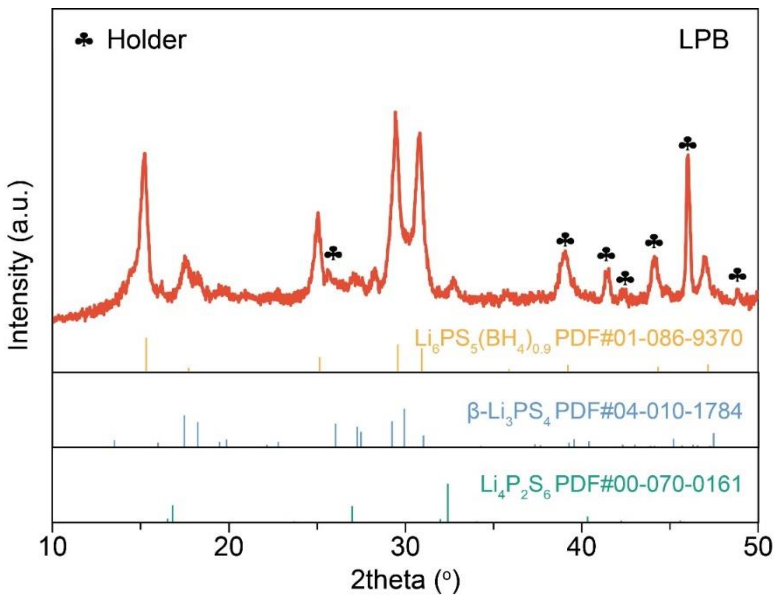

Supplementary Figure 12. XRD patterns of LPB referenced to the PDF cards of $Li_6PS_5(BH_4)_{0.9}$, β-Li_3PS_4, and $Li_4P_2S_6$.

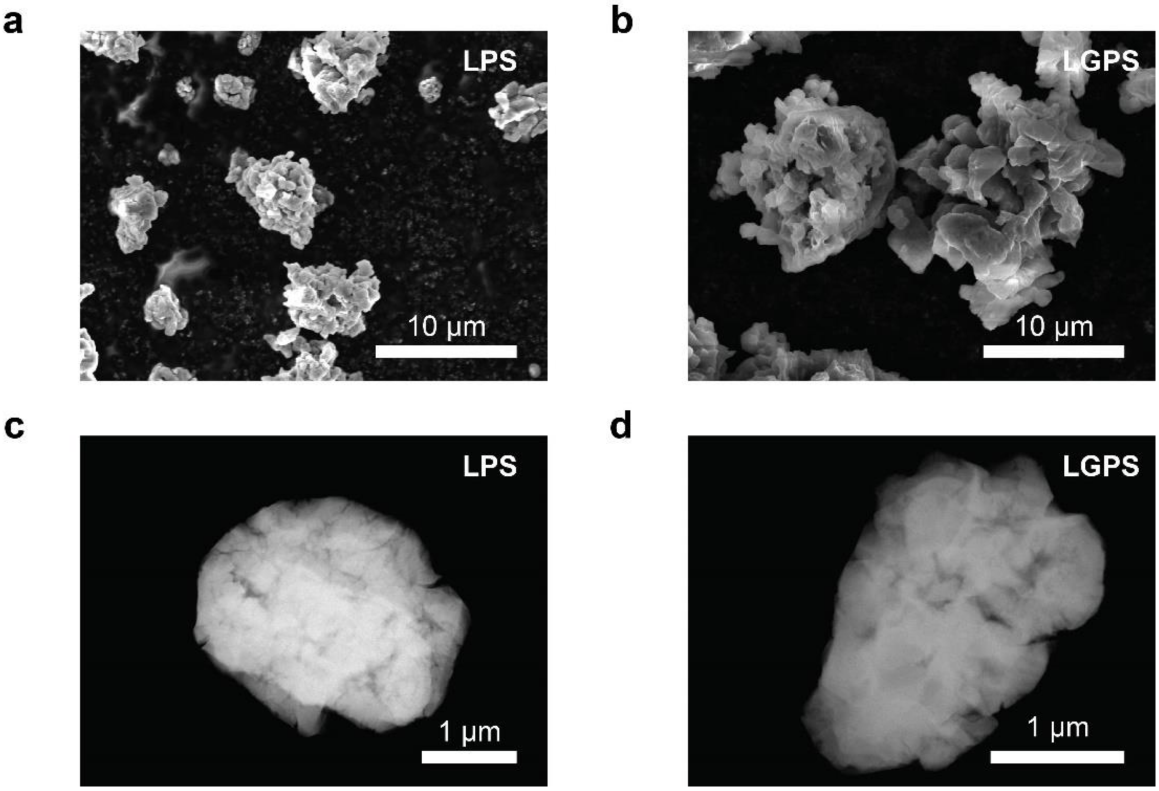

Supplementary Figure 13. SEM images of (**a**) β-Li_3PS_4 (LPS) and (**b**) $Li_{10}GeP_2S_{12}$ (LGPS). STEM images of (**c**) LPS and (**d**) LGPS. The size of LPS and LGPS primary particles is ~ 1 μm.

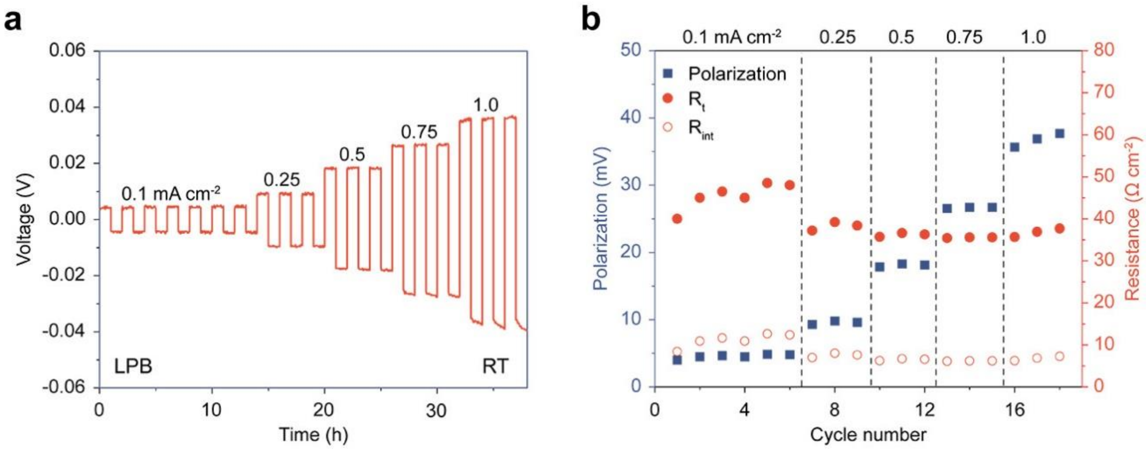

Supplementary Figure 14. (**a**) Cycling of a Li|LPB|Li symmetric cell at different current densities under 25 ^{o}C. (**b**) Evolution of voltage polarization, total resistance, and interfacial resistance during cycling. Calculation of the resistances is illustrated in Supplementary Note 3.

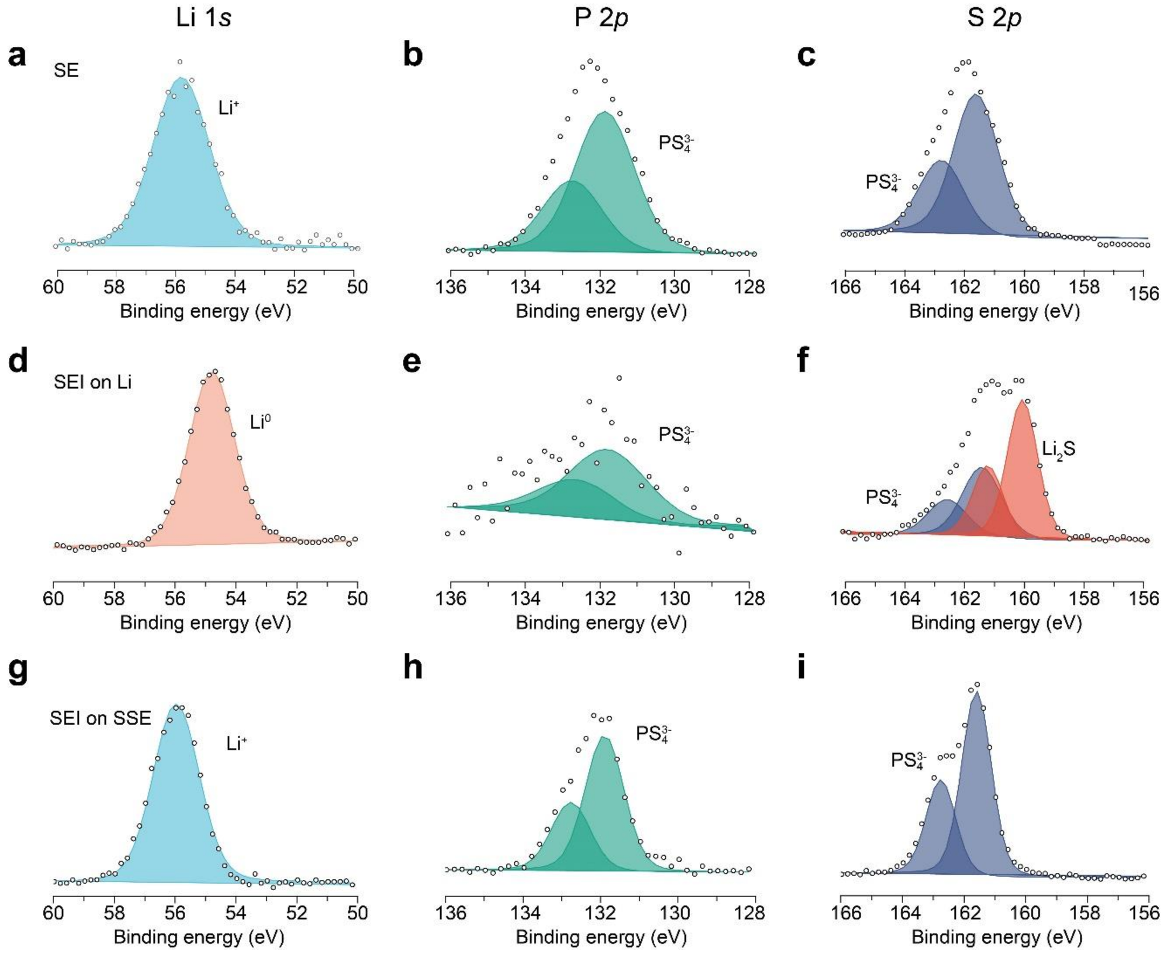

Supplementary Figure 15. X-ray photoelectron spectroscopy (XPS) spectra of (**a-c**) LPB SE, (**d-i**) solid electrolyte interphase (SEI) layer on the (**d-f**) Li metal surface, and (**g-i**) SE pellet surface at the Li/LPB interface.

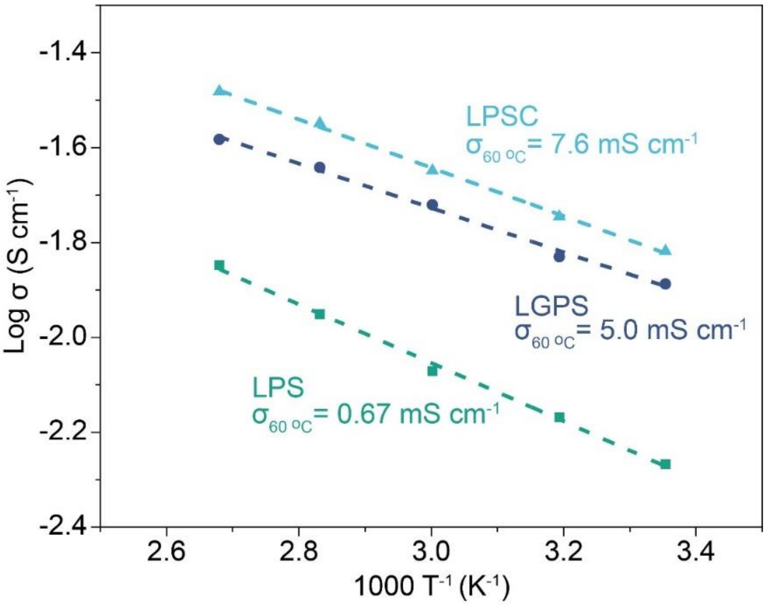

Supplementary Figure 16. The Arrhenius plots of LPS, LGPS, and Li_6PS_5Cl (LPSC) from 25 to 100 ^{o}C. The relative density of LPS pellet, LGPS pellet, and LPSC pellet is ~ 86% (1.57 g cm^{-3}, bulk density), ~ 87% (1.78 g cm^{-3}, bulk density), and ~ 92 % (1.64 g cm^{-3}, bulk density), respectively.

At 60 ^{o}C, LPB shows the highest ionic conductivity, LGPS exhibits the second highest ionic conductivity, and LPS possesses the lowest ionic conductivity. The discharge performances of cells with sulfur cathodes (i.e., S-C-LPB, S-C-LGPS, and S-C-LPS) do not enhance as the ionic conductivity of SE increases.

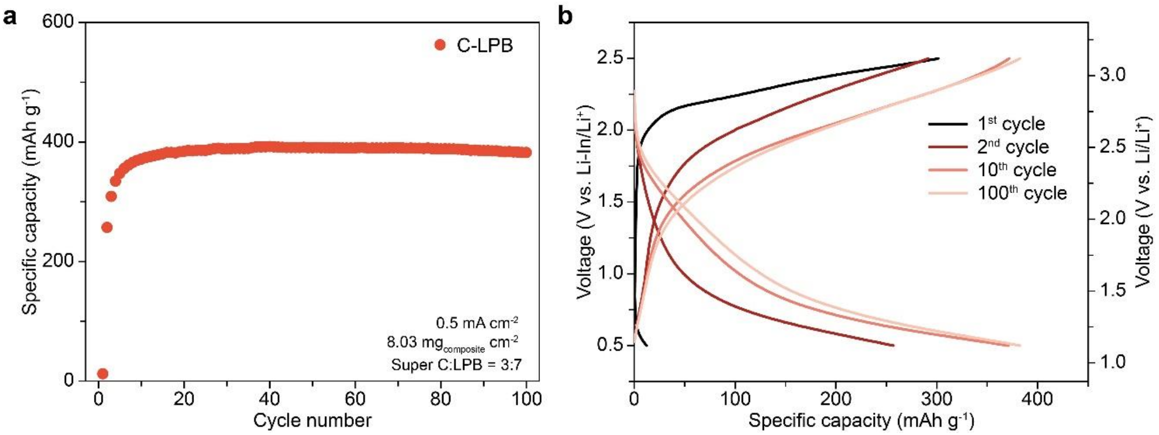

Supplementary Figure 17. (**a**) Cycling performance of a Li-In|LPB|C-LPB cell at 0.5 mA cm^{-2} and (**b**) corresponding voltage profiles at the 1st, 2nd, $10^{th,}$ and 100th cycles. The cell was evaluated between 0.5 and 2.5 V at 60 ^{o}C. The loading of the cathode is 8.03 mg $cm^{-2,}$ and the specific capacity is based on the weight of LPB.

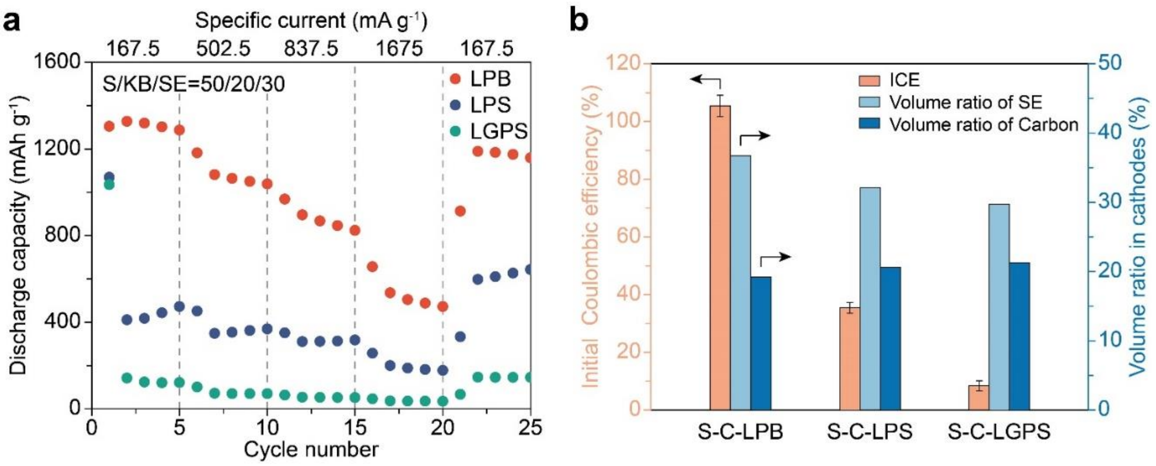

Supplementary Figure 18. (**a**) Rate performance of Li-In|LPB|S-C-SE cells having positive electrodes with 50 wt% of sulfur (KB/S/SE=20/50/30, w/w/w) using different SEs under 167.5, 502.5, 837.5, and 1675 mA g^{-1}. (**b**) Comparison of initial Coulombic efficiency (ICE, charge capacity/discharge capacity) and volume ratios of SE and carbon for different cathodes. All cells were tested between 0.5 and 2.5 V vs. Li-In/Li^+ at 60 ^oC.

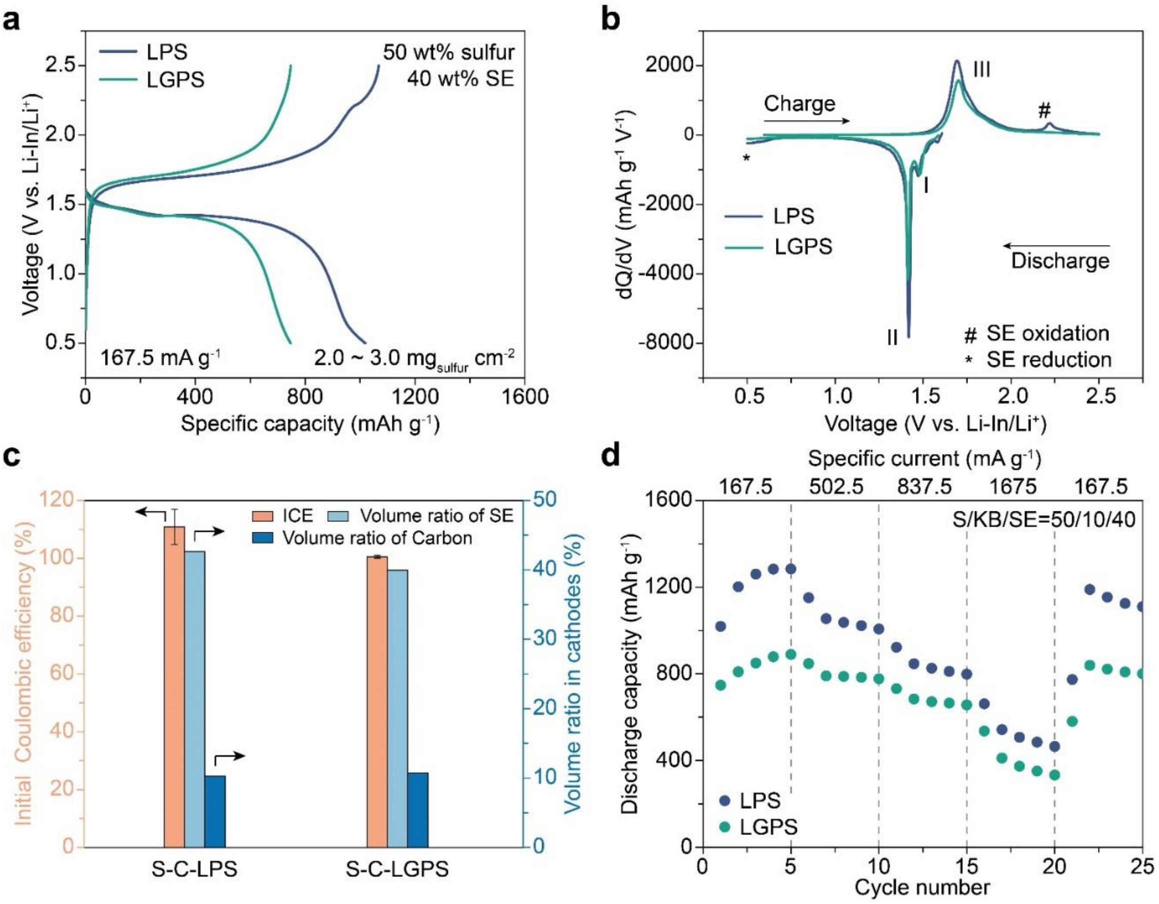

Supplementary Figure 19. (**a**) Galvanostatic discharge-charge curves of Li-In|LPB|S-C-SE cells having a positive electrode with 50 wt% sulfur and 40 wt% LPS or LGPS upon 167.5 mA g^{-1} at 60 ^{o}C, and (**b**) corresponding differential capacity vs. voltage (dQ/dV) curves. (**c**) Comparison of initial Coulombic efficiency (ICE, charge capacity/discharge capacity) and volume ratios of SE and carbon for different cathodes. (**e**) Rate performance of cells having positive electrodes with 50 wt% of sulfur (KB/S/LPB=20/50/30, w/w/w) using LPS or LGPS under 167.5, 502.5, 837.5, and 1675 mA g^{-1}. All cells were tested between 0.5 and 2.5 V vs. Li-In/Li^+ at 60 ^{o}C.

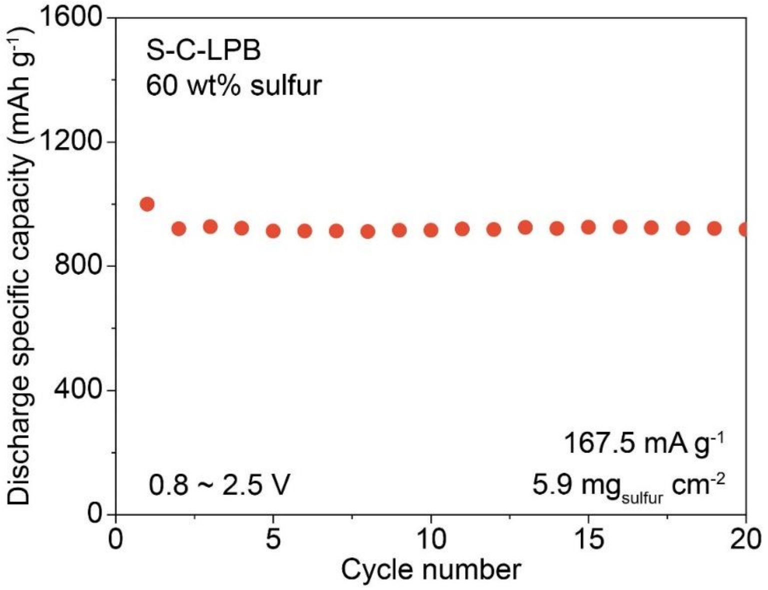

Supplementary Figure 20. Cycling performance of the Li-In|LPB|S-C-LPB cell with high areal sulfur loading of 5.9 mg cm^{-2} under 167.5 mA g^{-1} at 60 ^{o}C.

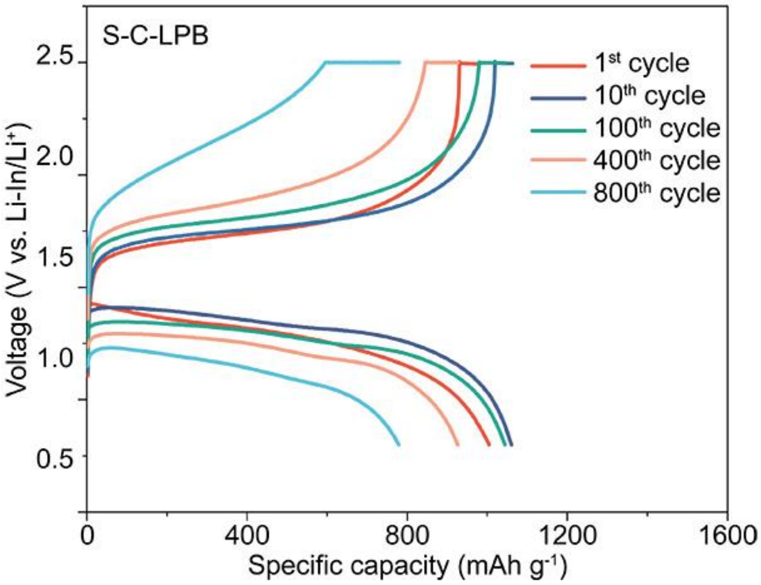

Supplementary Figure 21. Galvanostatic discharge-charge curves of the Li-In|LPB|S-C-LPB cell cycled at the 1st, 10th, 100th, 400th, and 800th cycle. The cell was cycled at 837.5 mA g^{-1} under CCCV mode (cutoff current, 167.5 mA g^{-1}; cutoff voltage, 2.5 V vs. Li-In/Li^+) between 0.8 and 2.5 V at 60 °C.

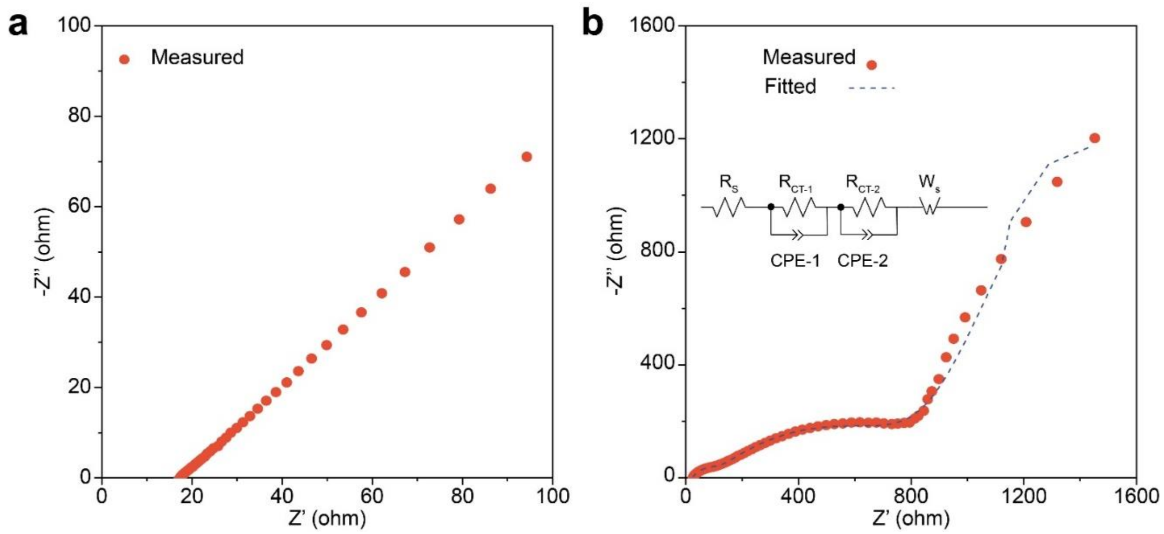

Supplementary Figure 22. Electrochemical impedance spectroscopy (EIS) spectra of the Li-In|LPB|S-C-LPB cell (**a**) before cycling and (**b**) after 1000 cycles. The numerical values of the fitted equivalent circuit elements are provided in Supplementary Table 4.

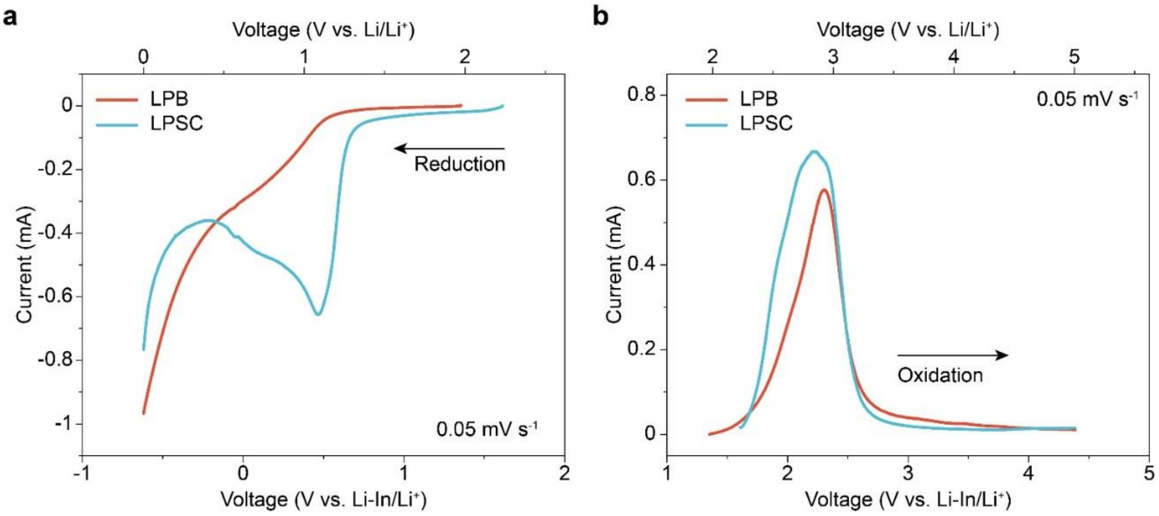

Supplementary Figure 23. Linear sweep voltammetry (LSV) curves of Li-In|LPB|C-LPB and Li-In|LPSC|C-LPSC (a) from open-circuit voltage to 0 V vs. Li/Li^+ (-0.62 V vs. $Li\text{-}In/Li^+$) and (b) from open-circuit voltage to 5 V vs. Li/Li^+ (4.38 V vs. $Li\text{-}In/Li^+$). The testing was performed at 60 ºC. The areal loadings of C-LPB and C-LPSC cathodes are between 3-4 mg cm^{-2}.

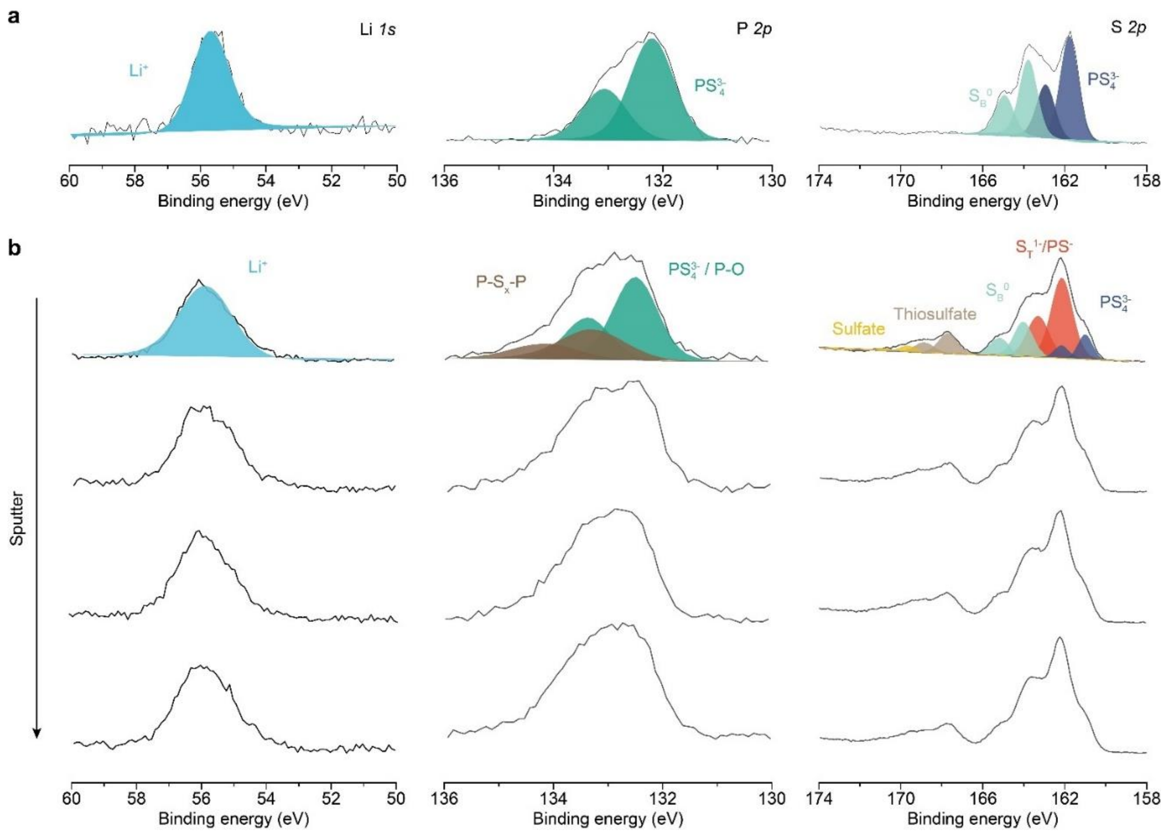

Supplementary Figure 24. (a) XPS spectra of pristine S-C-LPB cathode and (b) XPS depth profiling spectra of the S-C-LPB cathode disassembled from a Li-In|LPB|S-C-LPB cell after being cycled for 1000 cycles under 837.5 mA g^{-1} (constant-current constant-voltage mode; cutoff current, 167.5 mA g^{-1}; cutoff voltage, 2.5 V) between 0.8 and 2.5 V at the fully charged state. The sputtering duration is 0, 60, 120, and 150 mins for the curves from top to bottom. The sputtering rate is approximately 10 nm min^{-1} for SiO_2. A detailed discussion of the XPS spectra can be found in Supplementary Note 6.

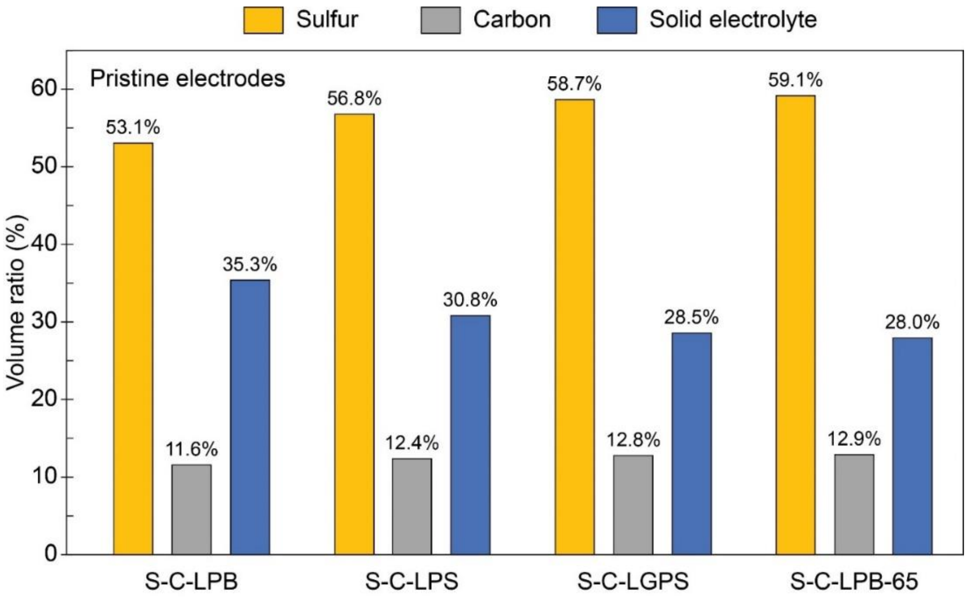

Supplementary Figure 25. Volumetric contents of cathode components in S-C-LPS, S-C-LPB, S-C-LGPS, and S-C-LPB-65. For estimation, we assumed the density of sulfur, carbon, LPB, LPS, and LGPS are 2.07, 1.491, 1.83, and 2.04 g cm^{-3}.

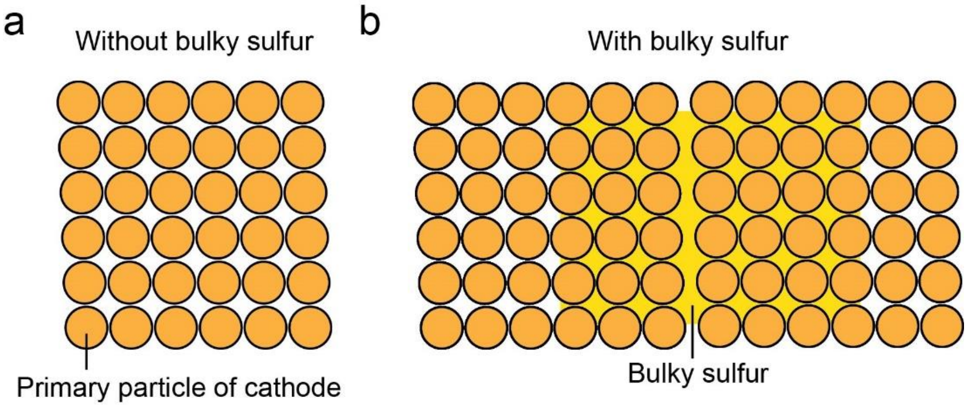

Supplementary Figure 26. Schematic illustration of bulky sulfur's influence on cathode powder aggregate size. The primary cathode particles are composed of uniformly distributed sulfur, SE, and carbon.

We speculate that the presence of micro-sized bulky sulfur particles could connect the primary particles together into big aggregate powders, leading to larger aggregate size of cathode powders. And the large aggregate powder size of the sulfur cathode composite may potentially indicate the presence of bulky inactive sulfur and poor electrochemical performance.

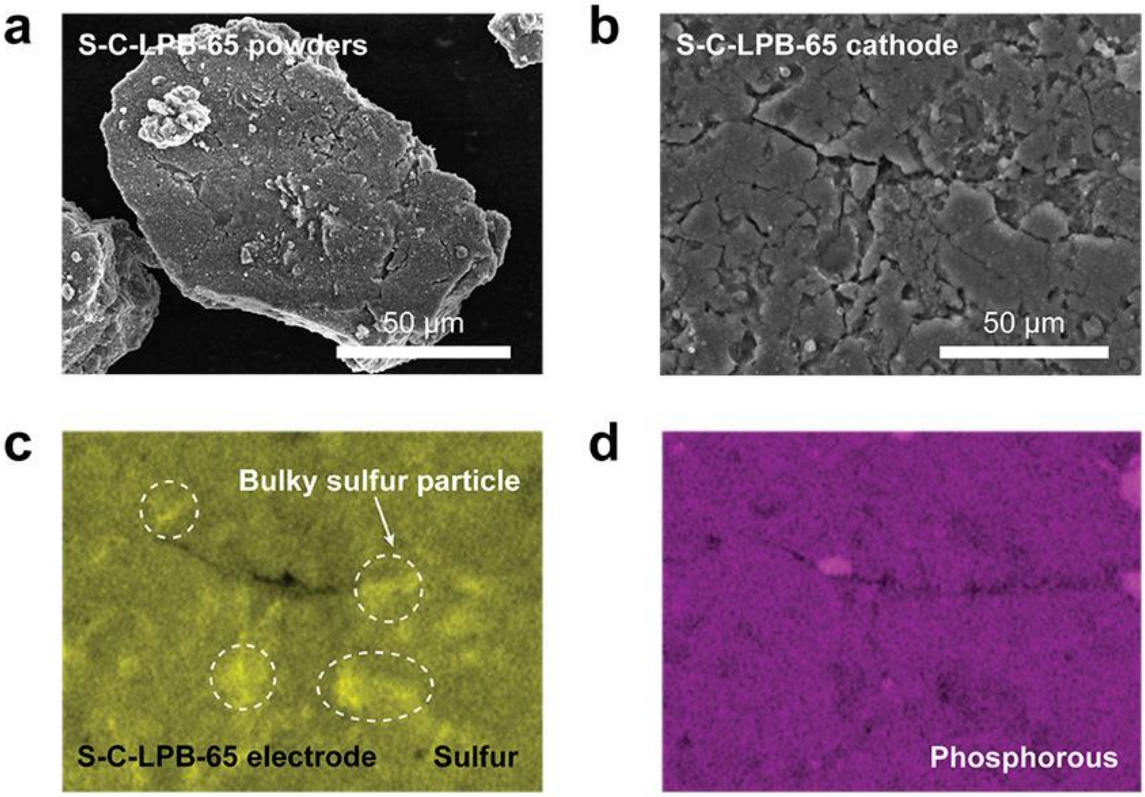

Supplementary Figure 27. a SEM images of S-C-LPB-65 powders. **b-d** SEM and corresponding EDS mapping images of pristine S-C-LPB-65 electrode.

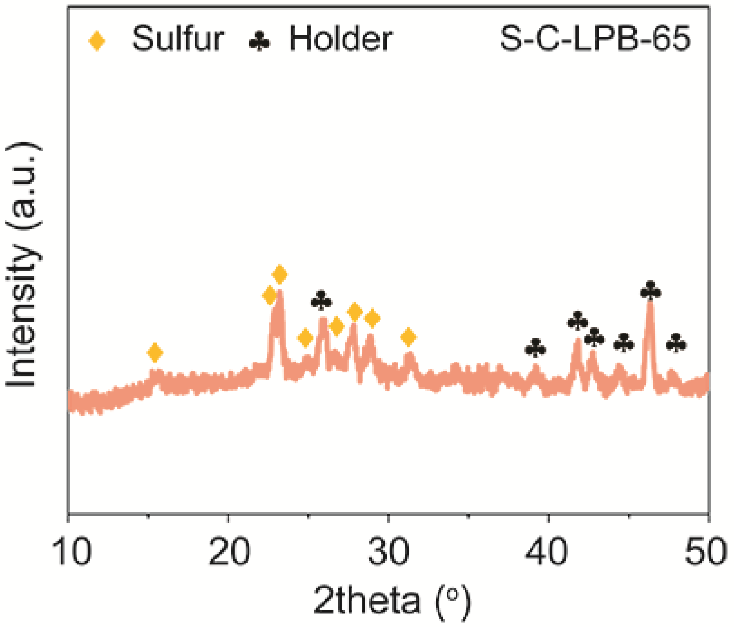

Supplementary Figure 28. XRD patterns of S-C-LPB-65 cathode powders.

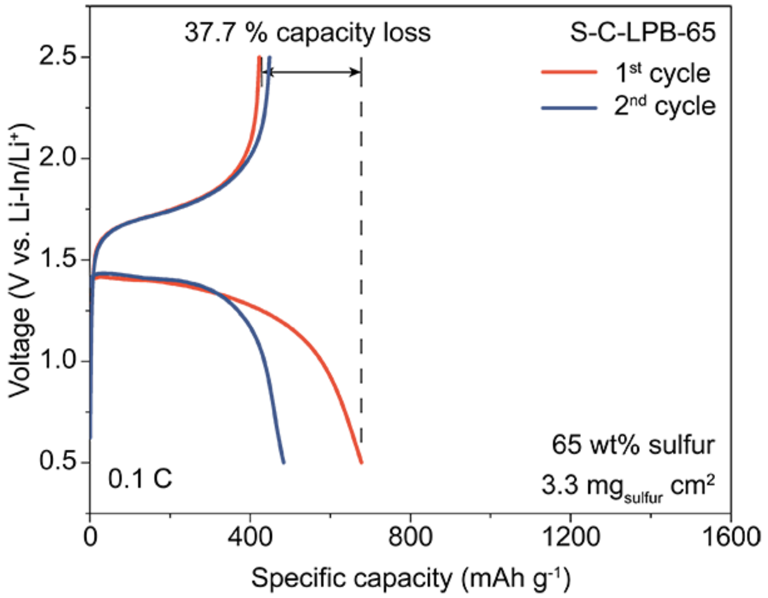

Supplementary Figure 29. Galvanostatic discharge-charge curves of Li-In|LPB|S-C-LPB-65 battery (S/KB/SE=50/10/17, w/w/w) at 167.5 mA g^{-1}. Testing was performed at 60 ºC.

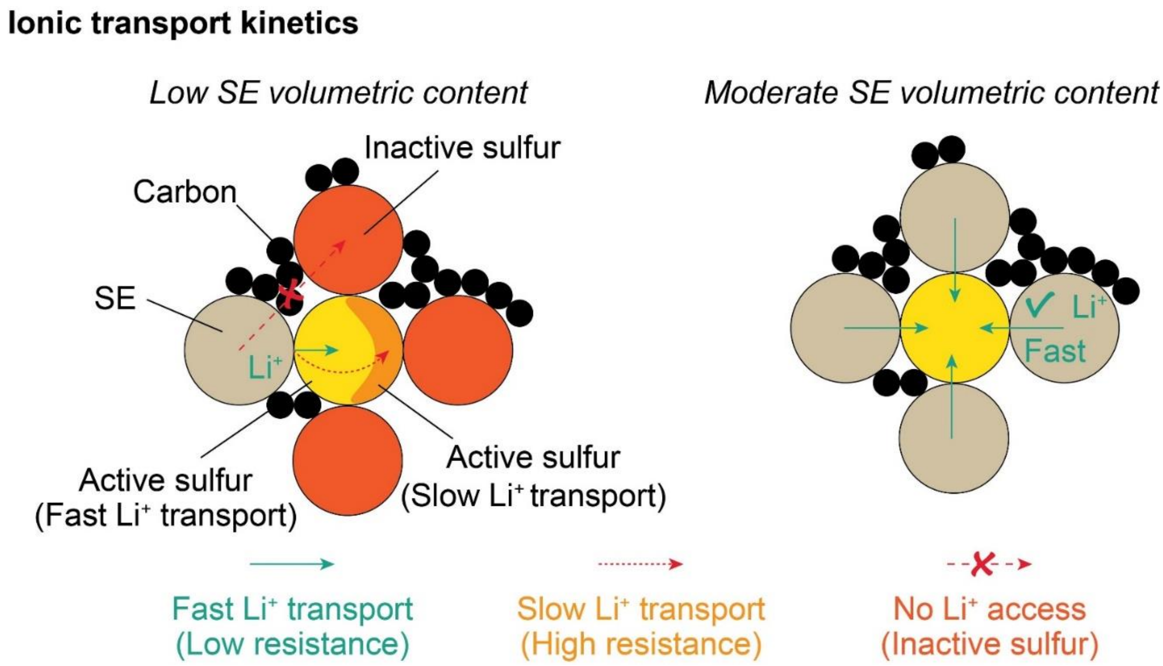

Supplementary Figure 30. Schematic illustration of the influence of SE's volumetric content on Li^+ transport kinetics and cathode resistance.

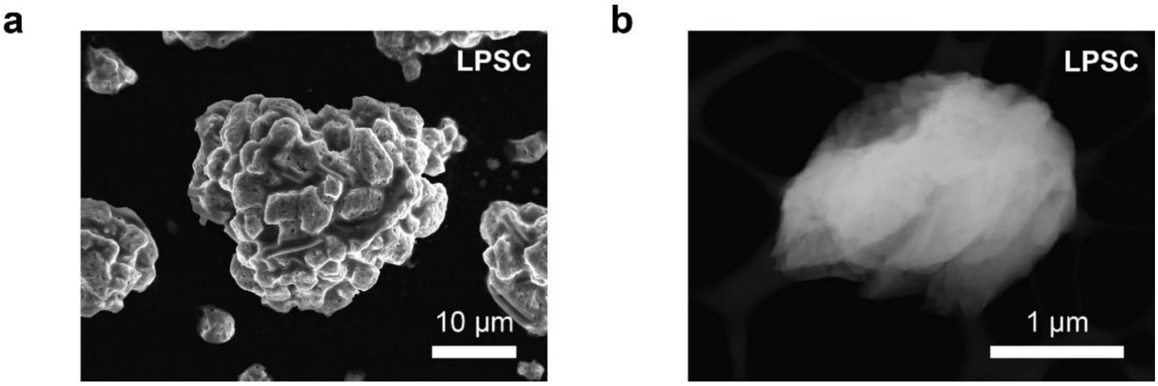

Supplementary Figure 31. (**a**) SEM and (**b**) STEM images of Li_6PS_5Cl (LPSC) SE.

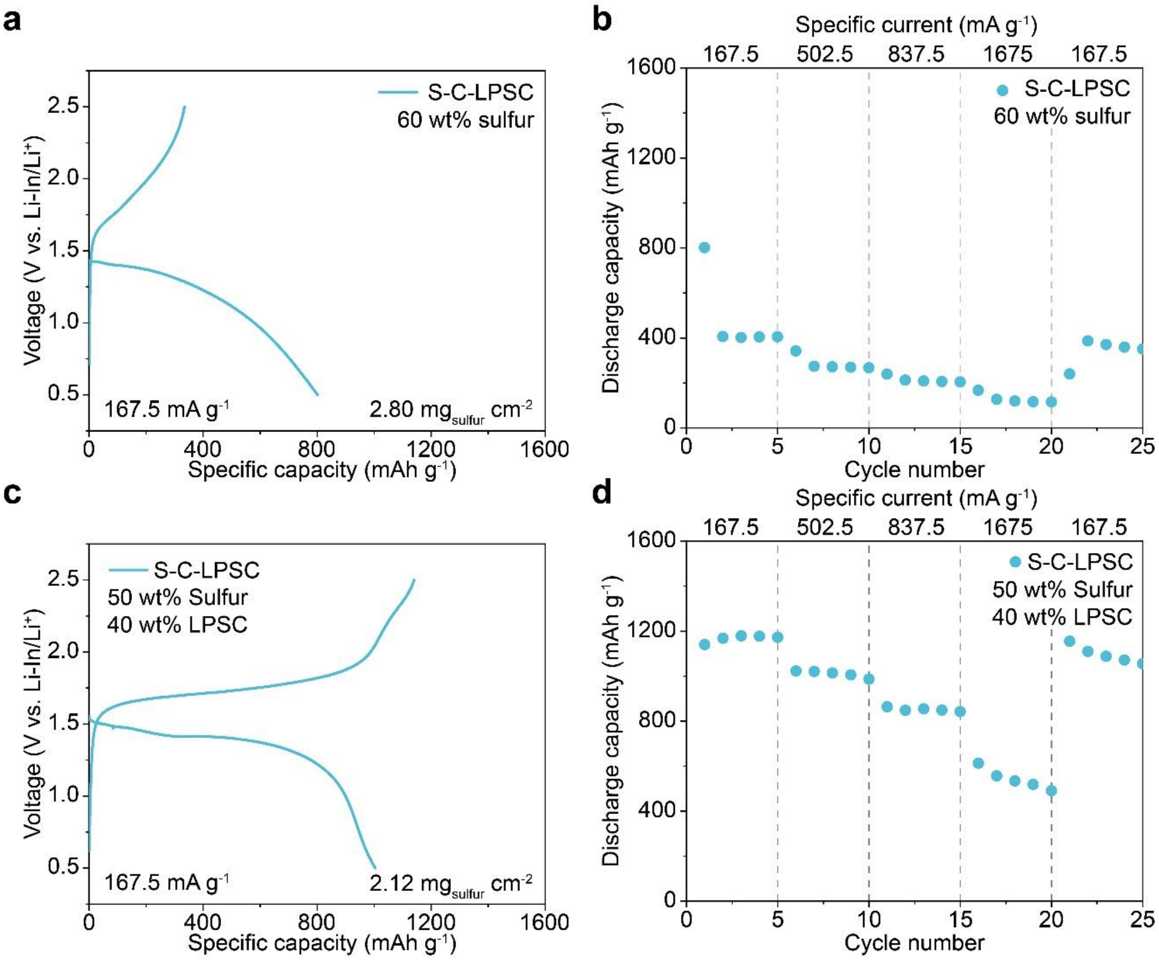

Supplementary Figure 32. **Electrochemical performance of Li-In|LPB|S-C-LPSC cells.** (**a**, **c**) Initial galvanostatic discharge-charge curves at 167.5 mA g^{-1} and (**c, d**) rate performance of cells using S-C-LPSC cathodes with 50 wt% of sulfur (**a**, **b**) and 40 wt% sulfur (**c**, **d**). All electrochemical testing was performed at 60 °C.

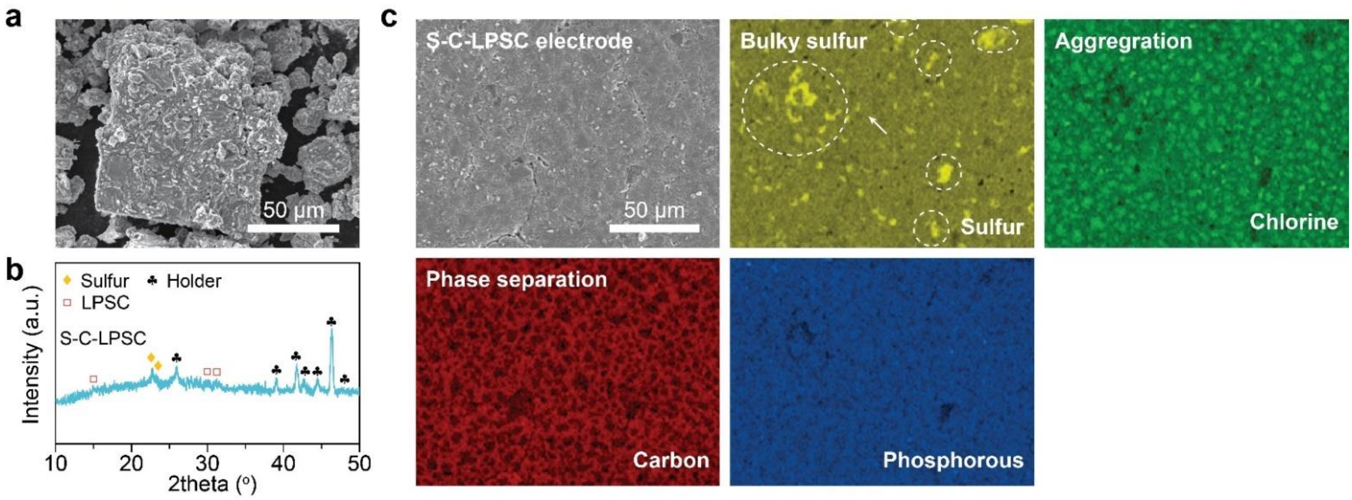

Supplementary Figure 33. (**a**) SEM image and (**b**) XRD of S-C-LPSC powders. (**c**) SEM and EDS mapping images of pristine S-C-LPSC cathode.

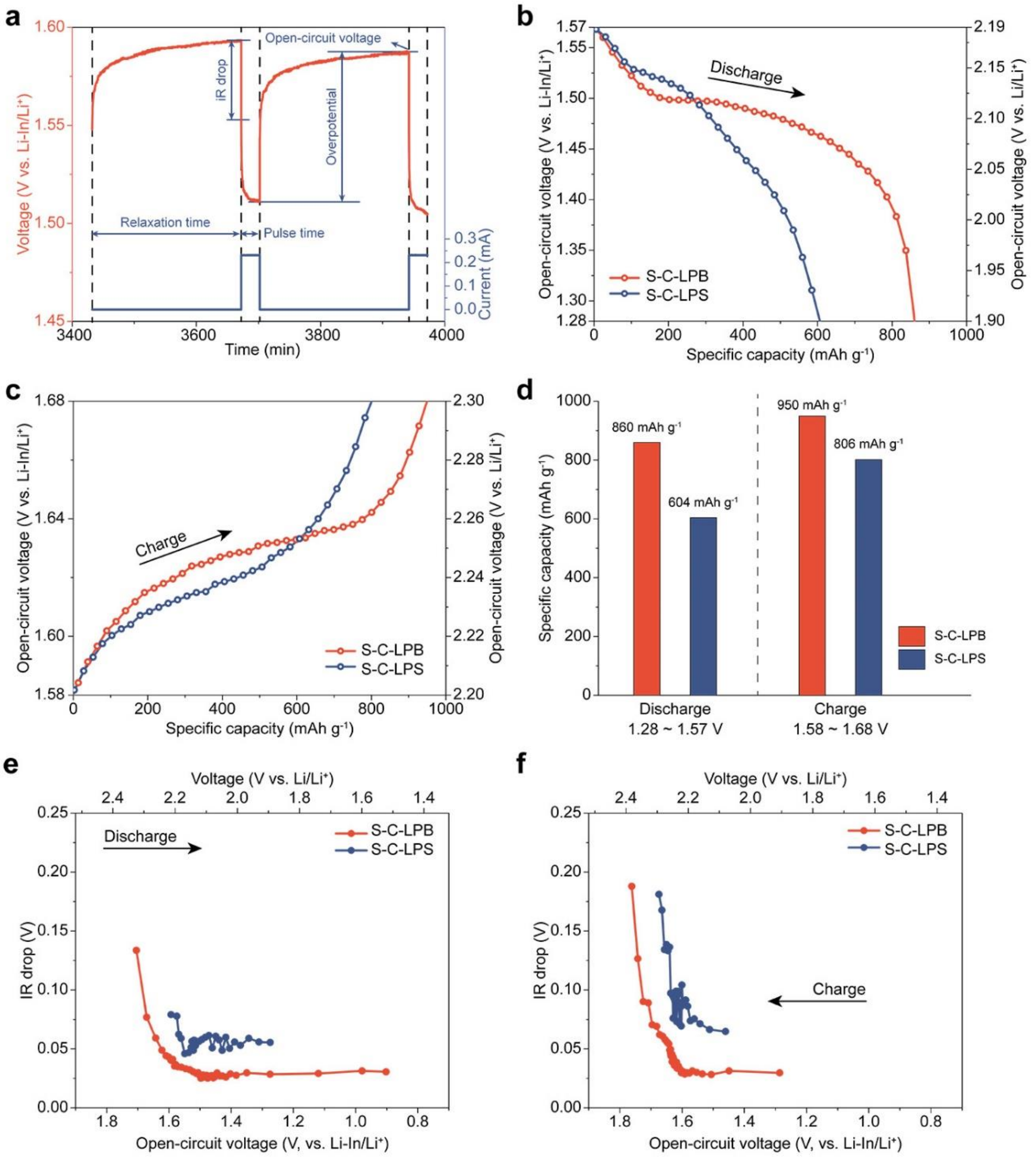

Supplementary Figure 34. Galvanostatic intermittent titration technique **(**GITT) analysis of Li-S ASSBs with S-C-LPB and S-C-LPS cathodes. (**a**) Schematic illustration of GITT curves at the selected steps during the discharging process. (**b-c**) Open-circuit voltage (OCV) profiles between selected voltage ranges of (**b**) 1.57 ~ 1.28 V during the discharging process and (**c**) 1.58 ~ 1.68 V during the charging process. The specific capacity at the initial OCV of the selected voltage windows was manually set to be 0 mAh g^{-1}. (**d**) Comparison of sulfur cathodes' specific capacities at the selected voltage ranges during lithiation/delithiation processes. (**e-f**) IR drop profiles of S-C-LPB ad S-C-LPS cathode during (**e**) the discharging process and (**f**) the charging process.

Supplementary Table 1. Summary of the density of different electrolytes for Li-S batteries.

Compositions	Phase	Density (g cm^{-3})	σ_{RT} (mS cm^{-1}) [a]
**Li_3PS_4-2$LiBH_4$ (This work)**	**Solid**	**1.491**	**6.09**
**1 M LiTFSI in DOL [b]:DME[c] (1:1, v/v)**	Liquid	1.13	11
**TTE [c]**	Liquid	1.533	6.5
**poly(ethylene oxide)**	Solid	1.2	0.6
**Li_3PS_4**	Solid	1.83	0.16
**$Li_{10}GeP_2S_{12}$**	Solid	2.04	10
**$Li_{1.3}Al_{0.3}Ti_{1.7}(PO_4)_3$**	Solid	2.92	0.213
**$Li_7La_3Zr_2O_{12}$**	Solid	5.108	0.2

[a] ionic conductivity at room temperature; [b] 1,3-dioxolane; [c] 1,2-Dimethoxyethane; [d] 1,1,2,2-tetrafluoroethyl-2,2,3,3-tetrafluoropropyl ether.

Supplementary Table 2. Summary of sulfide SEs synthesized through liquid-phase synthesis.

Compositions	Precursors	Solvent	T (ºC) [a]	σ_{RT} (mS cm^{-1}) [b]	Refs
**β-Li_3PS_4**	Li_2S, P_2S_5	THF	140	0.16	9
**β-Li_3PS_4**	Li_2S, P_2S_5	EA[d]	160	0.33	10
**β-Li_3PS_4**	Li_2S, P_2S_5	ACN[e]	200	0.12	11
**β-Li_3PS_4**	Li_2S, P_2S_5, $LiSC_2H_5$	THF	140	0.132	12
**Li_3PS_4**	Li_2S, P_2S_5	BA[f]	100	0.509	12
**$Li_7P_3S_{11}$**	Li_2S, P_2S_5	ACN	220	1.5	13, 14
**$Li_7P_3S_{11}$**	Li_2S, P_2S_5	DME[g]	250	0.27	15
**$Li_7P_3S_{11}$**	Li_2S, P_2S_5	ACN	250	0.97	16
**$Li_7P_3S_{11}$**	Li_2S, P_2S_5	ACN	260	1.5	17
**$Li_7P_2S_8I$**	Li_2S, P_2S_5, LiI	ACN	200	0.63	18
**$Li_7P_2S_8I$**	Li_2S, P_2S_5, LiI	EP	170	0.46	19
**Li_4PS_4I**	Li_2S, P_2S_5, LiI	DME	200	0.12	20
**Li_6PS_5Cl**	Li_2S, P_2S_5, LiCl	EA	550	1.1	21
**Li_6PS_5Cl**	Li_6PS_5Cl-BM	Ethanol	80	0.014	22
**Li_6PS_5Cl**	Li_6PS_5Cl-BM	Ethanol	180	0.19	23
**Li_6PS_5Cl**	Li_2S, P_2S_5, LiCl	ACN-ethanol	180	0.6	24
**Li_6PS_5Br**	Li_2S, P_2S_5, LiBr	THF-ethanol	550	3.1	25
**Li_6PS_5Br**	Li_2S, P_2S_5, LiCl	EP-ethanol	180	0.034	26
**Li_6PS_5Cl**	Li_2S, P_2S_5, LiCl	THF-ethanol	180	2.4	27
**$Li_6PS_5Cl_{0.5}Br_{0.5}$**	Li_2S, P_2S_5, LiCl, LiBr	THF-Ethanol	550	3.9	27
**$Li_{5.5}PS_{4.5}Cl_{1.5}$**	Li_2S, P_2S_5, LiCl	THF-Ethanol	550	3.9	27
**Li_6PS_5Cl**	Li_2S, P_2S_5, LiCl	Anisole-Ethanol	550	2.1	28
**Li_4SnS_4**	Li_2S, SnS_2	Methanol	200	0.09	29
**Li_4SnS_4**	Li_2S, SnS_2	Water	320	0.14	29
**$0.6Li_4SnS_4$-$0.4LiI$**	Li_2S, SnS_2, LiI	Methanol	299	0.41	30
**$Li_{10}GeP_2S_{12}$**	Li_2S, P_2S_5, GeS_2,	Hydrazine	240	0.113	31
**$Li_{6.5}P_{0.5}Ge_{0.5}S_5I$**	Li_2S, P_2S_5, GeS_2, LiI	Ethanol	180	0.54	30
**Li_3PS_4-$2LiBH_4$**	**Li_2S, P_2S_5, $LiBH_4$**	**THF[c]**	**160**	**6.09**	**This work**

[a] annealing temperature; [b] ionic conductivity at room temperature; [c] tetrahydrofuran; [d] ethyl acetate; [e] acetonitrile; [f] butyl acetate; [g] 1,2-dimethoxyethane.

Supplementary Table 3. Summary of the Li-S ASSBs performance in reported literature.

**No.**	**Cell configuration**	**ω_{sulfur} a) (wt%)**	**m_{Sulfur} b) (mg cm^{-2})**	**J c) (mA cm^{-2})**	**Specific capacity (mAh g^{-1})**	**Cycle number**	**T d) (^oC)**	**Refs**
**1**	Li|Li_3PS_4|S	30	0.75	0.025	1600	10	RM	8
**2**	Li|$75Li_2S$-$24P_2S_5$-P_2O_5|$Li_{10}GeP_2S_{12}$|S	12	0.4-0.5	0.42	1077	750	60	6
**3**	Li-In|Li_3PS_4-LiI|S	15	2.5	0.0835	710	20	RM	7
**4**	Li|Li_3PS_4|SeS_x	40	1.6	0.64	970	100	60	1
**5**	Li|Li_3PS_4·0.5LiI|S	35	2.73	2.28	1070	90	60	2
**6**	Li-In|Li_3PS_4|S	20	1.9	0.27	1444	200	RM	5
**7**	Li|$Li_{10}GeP_2S_{12}$|S	13.2	0.462	0.077	1193.3	400	60	3
**8**	Li-In|$Li_{10}GeP_2S_{12}$|S	25	1.1	0.18	1270	100	RM e)	4
**9**	**Li-In|LPB|S**	**60**	**2.57**	**2.15**	**1004.6**	**1000**	**60**	**This work**

a) sulfur content: b) sulfur mass loading; c) current density; d) testing temperature; e) room temperature.

Supplementary Table 4. Fitting parameters for the EIS spectra of the Li-S ASSB with the S-C-LPB cathode after 1000 cycles.

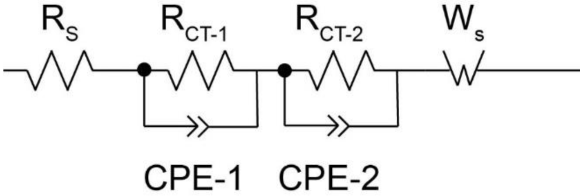

Parameters	Fitted values	Error	Error (%)
R_s (ohm)	23.87	0.34196	1.4326
R_{CT-1} (ohm)	76.18	5.5316	7.2612
CPE1-T (F s^{P-1})	1.04E-05	2.06E-06	19.782
CPE1-P	0.68445	0.019495	2.8483
R_{CT-2} (ohm)	782.3	24.602	3.1448
CPE2-T (F s^{P-1})	0.00027233	1.27E-05	4.6458
CPE2-P	0.50789	0.012968	2.5533
W_s-R (ohm)	5376	172.48	3.2083
W_s-T (s)	100	6.3335	6.3335
W_s-P	0.76653	0.014512	1.8932

Supplementary Note 1. Estimation of the specific energy of Li-S ASSBs.

The specific energy of Li-S ASSBs is estimated using the following parameters:

1. Sulfur cathode
   1) The volumetric content of sulfur is 50 vol%.
   2) The weight percent of sulfur in the cathode is *w* wt%.
   3) The volumetric carbon content in the cathode is fixed to be 15 vol%.
   4) The density of SSE is ρ_{SSE}
   5) The specific discharge capacity of the sulfur cathode is 1000 mAh g^{-1}.
   6) The average voltage during discharge is 2.0 V.
   7) Areal sulfur loading is m g_{sulfur} cm^{-2}.
2. Membrane
   1) The thickness of the membrane is 25 μm.
3. Anode
   1) The areal capacity ratio of the negative electrode to the positive electrode (N/P) is 2.
4. Current collectors
   1) The areal weight of current collectors (Cu and Al) is 5.83 mg cm^{-2}, which is estimated based on the double-sided electrode cell design.

Next, the cell-level specific energy of Li-S ASSBs could be estimated using the following equation:

$$\text{Specific energy}=\frac{1000\text{ mAh g}^{-1}\times m\text{ g}_{\text{sulfur}}\text{ cm}^{-2}\times 2.0\text{ V}}{0.0025\text{ cm}\times\rho_{SSE}\text{ g cm}^{-3}+\frac{2\times 1000\text{ mAh g}^{-1}\times m\text{ g}_{\text{sulfur}}\text{ cm}^{-2}}{3860\text{ mAh g}^{-1}}+\frac{m\text{ g}_{\text{sulfur}}\text{ cm}^{-2}}{\text{w }\%}+0.00583\text{ g cm}^{-2}}$$

The weight percent of sulfur in the cathode could be calculated using the following equation:

$$w\ \%=\frac{2.07\text{ g cm}^{-3}\times 50\text{ vol\%}}{2.07\text{ g cm}^{-3}\times 50\text{ vol\%}+1.9\text{ g cm}^{-3}\times 15\text{ vol\%}+\rho_{SSE}\times(100\text{-}50\text{-}15)\text{ vol\%}}$$

With different SE densities and areal sulfur loading, the specific energy of Li-S ASSBs and sulfur content (wt%) in the cathode can be calculated, and the results are depicted in Figure 1b and Supplementary Figure 4.

Supplementary Note 2. Crystal phase composition in LPB.

Compared with solid-phase synthesized sulfide SEs, liquid-phase synthesized sulfide SEs usually have more impurities due to the side reactions of precursors with organic solvents, making the determination of the detailed composition of SE crystals challenging. In previous literature, the molar ratios of added precursors are typically used to determine the composition of the formed crystals synthesized via the liquid-phase method[9,18,27]. According to the molar ratios of the precursors used for synthesizing LPB ($Li_2S/P_2S_5/LiBH_4$=3/1/4), the composition of the formed crystal should be $Li_5PS_4(BH_4)_2$. However, considering the presence of impurities, the actual composition may differ from the calculated formula. Although the XRD patterns LPB share similar peak position and relative peak intensity with previously reported XRD patterns of $Li_6PS_5BH_4$[32], we believe it is inaccurate to assign the liquid phase synthesized crystal to be $Li_6PS_5BH_4$ argyrodite. Therefore, we use $Li_{6-x}PS_{5-x}(BH_4)_{1+x}$ ($-1 \leq x \leq 1$) to represent the crystal composition of LPB.

Supplementary Note 3. Electrochemical stability of LPB against lithium metal anodes.

Furthermore, we also examined the electrochemical stability of LPB against lithium metal using a Li|LPB|Li symmetric cell. The cell was assembled using a hot-pressed LPB pellet and cycled at different current densities from 0.1 mA cm^{-2} to 1.0 mA cm^{-2} under 6 ~ 8 MPa at room temperature. The voltage profile as a function of time is shown in Supplementary Figure 14a. The cell cycled stably without short circuit failure until the current density reached above 1 mA cm^{-2}, demonstrating that the critical current density is 1 mA cm^{-2}. To gain an in-depth understanding of the evolution of the SEI layer, both areal total resistance (R_t) and areal interfacial resistance (R_{int}) were calculated using the following equations:

$$R_t=\frac{U}{J} \qquad (1)$$

$$R_{pellet}=\frac{l}{\sigma_{25℃}} \qquad (2)$$

$$R_{int}=\frac{R_t\text{-}R_{pellet}}{2} \qquad (3)$$

where U is the voltage polarization, J is the current density, R_{pellet} is the areal bulk resistance contributed by the electrolyte pellet, l is the thickness of the pellet, and $\sigma_{25℃}$ is the measured ionic conductivity of LPB at 25 ^{o}C. The calculated resistance and the voltage polarization are illustrated in Supplementary Figure 14b. During the first few cycles at 0.1 mA cm^{-2}, the voltage polarization, total resistance, and interfacial resistance gradually increased due to the growth of the SEI layer. After the current density increased to 0.25 mA cm^{-2}, the evolution of the SEI layer stabilized with a low interfacial resistance of 6 ~ 7 Ω cm^2, which remains almost constant even at 1 mA cm^{-2}. This result demonstrates the formation of a low-resistance metastable SEI layer at the Li/LPB interface.

Further analysis of the SEI layer by XPS reveals that this low-resistance SEI layer is mainly composed of Li_2S. High-resolution XPS spectra of Li 1*s*, P 2*p*, and S 2*p* were collected for the original LPB SE (Supplementary Figure 15a-c) and the lithium/SE interface at both lithium metal (Supplementary Figure 15d-f) and the SE (Supplementary Figure 15h-j) surfaces. On the surface of the SE pellet, with peaks sitting at their original position, there is no sign of LPB's decomposition. On the lithium metal surface, a peak corresponding to Li^0 of Li metal was observed at ~ 54.8 eV in Li 1*s* spectra, distinct from the Li 1*s* spectra of LPB SE, with the peak sitting at ~ 56 eV. In the P 2*p* spectra of SEI on Li, only one pair of doublet peaks attributed to LPB SE were observed at ~ 131.8 and ~ 132.6 eV with weak intensity, while any peak associated with Li_3P from the reduction of the SE was not observed. In the S 2*p* spectra of SEI on Li, the formation of Li_2S was confirmed by the presence of doublet peaks of Li_2S at ~ 160 and ~ 161 eV. Together, the results

demonstrated the formation of a Li_2S-rich metastable SEI layer at the Li/LPB interface, making LPB stable against the lithium metal anode.

Supplementary Note 4. Calculation of the specific energy and volumetric energy density of Li-S ASSBs （based on the weight/volume of the sulfur cathode） .

1. The estimation of sulfur cathodes' specific energy is based on the following parameters:
   1) The weight percent of sulfur in the cathode is *w* wt%.
   2) The specific discharge capacity of the sulfur cathode is *a* mAh g^{-1}.
   3) For the S-C-LPB cathode, we used the average discharge voltage of 1.935 V (vs. Li/Li^{+}) for estimation.
   4) Areal cathode loading is *m* mg cm^{-2}.

Next, based on the above parameters, the specific energy of sulfur cathodes could be estimated using the following equation:

$$\text{Specific energy} = \frac{m\ \text{mg cm}^{-2} \times w\% \times a\ \text{mAh g}^{-1} \times 1.935\ \text{V}}{m\ \text{mg cm}^{-2}} \times \frac{1\ \text{Ah}}{1000\ \text{mAh}} \times \frac{1000\ \text{g}}{1\ \text{kg}} = 2aw\%\ \text{Wh kg}^{-1}$$

2. The energy density of sulfur cathodes can be similarly calculated based on the following parameters:
   1) Areal mass loading of sulfur, carbon and solid electrolyte of the cathode are $m_S, m_C,$ and m_{SE}, respectively. $m_S/m_C/m_{SE}$=50/20/24.
   2) The specific discharge capacity of the sulfur cathode is *a* mAh g^{-1}.
   3) For the S-C-LPB cathode, we used the average discharge voltage of 1.935 V (vs. Li/Li^{+}) for estimation.
   4) The porosity of the cathode is 95%.
   5) The density of sulfur, carbon and solid electrolyte in the cathode are $\rho_S, \rho_C,$ and ρ_{SE}, respectively. ρ_S=2.07 g cm^{-3}, ρ_C=1.9 g cm^{-3}, and ρ_{SE}=1.491 g cm^{-3} for LPB.

$$\text{Energy density} = \frac{a\ \text{mAh g}^{-1} \times m_S\ \text{mg cm}^{-2} \times 1.935\ \text{V}}{\left(\frac{m_C}{\rho_C} + \frac{m_S}{\rho_S} + \frac{m_{SE}}{\rho_{SE}}\right) \div 95\%} \times \frac{1\ \text{Ah}}{1000\ \text{mAh}} \times \frac{1000\ \text{ml}}{1\ \text{L}}$$

Supplementary Note 5. Capacity contribution from LPB.

LPB, like other sulfide-based SEs, is electrochemical redox active and can contribute to capacity during charge/discharge cycles in the S-C-LPB cathodes (Supplementary Figure 17). Here, we further discuss the capacity contribution from the LPB redox reaction in the S-C-LPB cathode (KB/S/SE=10/50/24, w/w/w) based on the data in Figure 3e. If we assume the LPB delivers the same discharge capacity as was measured in Supplementary 17, the discharge capacity contribution of LPB is ~ 5.8 mAh g^{-1}_{sulfur} (~ 12.1 mAh g^{-1}_{LPB}) at the first cycle and < 187.7 mAh g^{-1}_{sulfur} in the following cycles. It shows that sulfur contributed to ~ 99.5 % discharge capacity at the first cycle and > 82% discharge capacity (based on the discharge capacity of the S-C-LPB at the last cycle in Figure 3e and LPB's maximum capacity contribution of 187.7 mAh g^{-1}_{sulfur} in the following cycles at 167.5 mA g^{-1}. Note that it is just a rough estimation. The capacity contribution of LPB may vary depending on the internal resistance of the sulfur cathode, the contacting area between carbon and LPB, and the current density applied. Considering the S-C-LPB cathode with a large amount of sulfur and a small amount of LPB & carbon, the actual capacity contribution from LPB might be smaller than the above-calculated values. Collectively, the results show that the good discharge capacity of S-C-LPB is mainly attributed to the high sulfur utilization.

Supplementary Note 6. Causes for the capacity decay of Li-S ASSBs during long-term cycling.

Two main factors contribute to the capacity fading of Li-S ASSBs using S-C-LPB cathode, namely, electrochemical and chemical degradation of LPB SE.

1. Electrochemical degradation of LPB SE.

Sulfide SEs typically possess a narrow electrochemical stability window between 2 ~ 2.5 V (vs. Li/Li^+).[33,34] LSV results in Supplementary Figure 23 illustrate that LPB with improved reduction stability than LPSC will still inevitably undergo electrochemical oxidation/reduction in the designated electrochemical voltage window of 0.8 ~ 2.5 V (vs. $Li\text{-}In/Li^+$, or 1.42 ~ 3.12 V vs. Li/Li^+). Luckily, within the voltage window, the lithiation/delithiation of LPB is relatively stable with minor capacity decay (Supplementary Figure 17), which explains the good cycling stability of the S-C-LPB cathode. However, it should be noted that after hundreds of cycles, the side effects caused by LPB degradation may accumulate and impede ion transport, giving rise to higher internal resistance and capacity decay.

In addition to the electrochemical characterization results, XPS analysis also confirms the oxidation of LPB. Within the adopted operation voltage, two main decomposition products are S^0/Li_2S and $P_2S_7^{4-}$. One possible exemplary oxidation reaction of LPB SE[34] is

$$2PS_4^{3-} \rightarrow P_2S_7^{4-} + S^0 + 2e^- \quad (1)$$

The formation of $P_2S_7^{4-}$ was confirmed by the observation of $P\text{-}S_x\text{-}P$ species in the P 2*p* XPS spectra (doublet peaks at 133.23 & 134.10 eV) of the cycled S-C-LPB cathode (Supplementary Figure 24). Meanwhile, the other decomposition product, S^0/Li_2S, is electrochemically active and thus contributes to the capacity increase in the first 20 cycles of the cell.

2. Chemical degradation of LPB SE.

Sulfide SEs are mostly chemically unstable against moisture.[35] Since our testing cell is not fully protected from moisture in the air, the chemical degradation of LPB SE may still occur. As a result, the chemically decomposed LPB SEs, forming sulfate species (Supplementary Figure 24, doublet peaks of 176.69 & 168.85 eV and 169.65 & 170.81 eV in S 2*p* spectra), also induce higher resistance and contribute to the continuous capacity fading of the Li-S ASSB. In addition, we noticed that the doublet peaks attributed to the LPB SE in P 2*p* spectra slightly shifted from 132.20 & 133.07 eV to 132.49 & 133.23 eV, which might be caused by the formation of the P-O bond.

Together, the degradation of LPB SE will impede ion transport and increase charge-transfer resistance (Supplementary Figure 22), leading to the continuous growth of voltage polarization (Supplementary Figure

21) and capacity decay. After 1000 cycles, even though no Li_2S was observed in the cathode at the charging state, the intensity of the bridging sulfur (S_B^0) peak significantly decreased. Instead, the peak corresponding to terminal sulfur (S_T^{1-})/P-S_x-P shows the highest intensity, possibly suggesting that lithium polysulfide (Li_2S_x) reaction intermediates were not fully oxidized to S_8 due to the large resistance of the cathode.

Supplementary Note 7. Influence of SE particle size on cathode performance.

The influence of SE particle size on $LiNi_{0.5}Mn_{0.3}Co_{0.2}O_2$ (NMC) cathode performance in all-solid-state lithium batteries has been reported in the literature, revealing that smaller SE particle size is beneficial for attaining sufficient ionic percolation pathways in the cathode.[36] Here, we additionally show that SE particle size will also influence sulfur cathode performance by regulating ionic transport pathway sufficiency.

Li_6PS_5Cl (LPSC) SE with high ionic conductivity (Supplementary Figure 16), low density (1.64 g cm^{-3}) but large particle size (Supplementary Figure 31) was employed for preparing the sulfur cathode with ~ 60 wt% of sulfur (S/KB/LPSC=50/10/24, denoted as S-C-LPSC). Despite LPSC's higher volume ratio (~ 33.2 vol%) and ionic conductivity than LPS (~ 30.8 vol%), the S-C-LPSC cathode demonstrated worse electrochemical performance than S-C-LPS cathodes with lower initial Coulombic efficiency (41.77 %) and smaller discharge specific capacities at all current rates (Supplementary Figure 32a, b, and Figure 3e). As shown in Supplementary Figure 32c, d, the sulfur cathode using LPSC SE with a large particle size can deliver good electrochemical performance only after enough LPSC is added (S/KB/LPSC=50/10/40, SE volume ratio of 45.3%). We speculate that the larger particle size of LPSC (particle size of 5~10 μm, Supplementary Figure 31) than that of LPS (Supplementary Figure 13) is causing the poor electrochemical performance of S-C-LPSC cathodes. Further analysis of the S-C-LPSC cathode powders and electrode via SEM-EDS and XRD revealed large S-C-LPSC aggregate powders and bulky inactive crystalline sulfur particles in the S-C-LPSC electrode (Supplementary Figure 33), similar to that of the S-C-LPS powders and electrode (Figure 4a-d), indicating poor content uniformity and insufficient Li-ion transport pathway. Intriguingly, in the meantime, we also observed phase separation between SE and carbon in the SEM-EDS images, possibly originating from the large particle size of LPSC. The phase separation, detrimental to ion/electron transport, shall further jeopardize the electrochemical performance of the cathode. Collectively, the results show that, besides the volume ratio of SE in the cathode, the large particle size of SE shall also compromise cathode content uniformity and induce the formation of inactive bulky sulfur, rendering insufficient Li-ion transport pathways and poor sulfur utilization. These results also highlight the uniqueness of the LPB SE developed via a novel liquid-phase method with low density and small particle size.

Supplementary Note 8. GITT results analysis

Assumptions:

a. The batteries reached thermodynamics equilibrium after each 4-hour resting.

b. The electrochemical reactions in both cathodes are identical at the same OCV.

1. Comparison of the amount of active sulfur (Supplementary Figure 34b-d)

The specific capacity of the S-C-LPS and S-C-LPB cathodes is quantified within certain OCV ranges during the lithiation/delithiation process. It allows us to exclude the kinetic contribution in specific capacity differences between the two sulfur cathodes and compare active sulfur amounts. We manually selected the OCV windows of 1.57 ~ 1.28 V (2.19 ~ 1.9 V vs. Li/Li^+) during lithiation and 1.58 ~ 1.68 V (2.20 ~ 2.30 V vs. Li/Li^+) during delithiation for calculation and comparison. 1.9 V was chosen because almost all the active sulfur was converted to Li_2S at this stage. The OCV profiles within the selected voltage windows are shown in Supplementary Figure 34b-c. The evolution trends of OCV curves in both cathodes are similar, especially at the beginning and end of the discharging/charging process, indicating similar electrochemical reactions at the same OCV. However, we also notice some disparities in the OCV profiles. Specifically, during the lithiation process between 1.55 and 1.50 V, the OCVs of the S-C-LPS cathode are slightly higher (< 20 mV) than that of the S-C-LPB cathode at the same discharge capacity. Meanwhile, during the delithiation process between 1.60 and 1.64 V, the OCVs of the cell with the S-C-LPS cathode are slightly lower (< 10 mV) than that of the cell with S-C-LPB cathode at the same charge capacity. We speculate that the redox reactions of SE or the tiny potential differences of Li-In anodes in the two batteries cause such minor differences in OCV profiles. However, despite such differences, we suppose the total specific capacity differences in the selected OCV windows (Supplementary Figure 34d) can still reflect the amount of electrochemically active sulfur in the two cathodes, considering the sufficiently large OCV voltage windows and the significantly higher specific capacity contribution from the active material than thiophosphate SE within the selected OCV ranges. Therefore, the result indicates more electrochemically active sulfur in the S-C-LPB cathode than in the S-C-LPS cathode.

2. Comparison of ion transport kinetics

The overpotential, which reflects the total resistance of the cell, and IR drop, which is related to the charge transfer resistance and uncompensated resistance, are monitored to compare the ion transport kinetics in S-C-LPB and S-C-LPS cathodes (Figure 4k and Supplementary Figure 34e-f). Since the anode and SE membrane are the same in both batteries, the differences in overpotential and IR drop are attributed to the resistance of

the sulfur cathodes. Therefore, the lower overpotential and IR drop in the Li-S ASSB with the S-C-LPB cathode suggest a lower resistance of the S-C-LPB cathode than that of the S-C-LPS cathode. Considering the higher ionic conductivity of LPB than LPS and less inactive bulky sulfur in the S-C-LPB cathode than in the S-C-LPS cathode (Figure 4i and Supplementary Figure 30), we deem that the better Li^+ transport kinetics in the S-C-LPB cathode contributes to its lower resistance.

Supplementary References

1. Li, X. *et al.* High-performance Li–SeS_x all-solid-state lithium batteries. *Adv. Mater.* **31**, 1808100 (2019).
2. Bonnick, P., Niitani, K., Nose, M., Suto, K., Arthur, T.S., Muldoon, J. A high performance all solid state lithium sulfur battery with lithium thiophosphate solid electrolyte. *J. Mater. Chem. A* **7**, 24173-24179 (2019).
3. Zhang, Q., Huang, N., Huang, Z., Cai, L., Wu, J., Yao, X. CNTs@S composite as cathode for all-solid-state lithium-sulfur batteries with ultralong cycle life. *J. Energy Chem.* **40**, 151-155 (2020).
4. Zhu, G-L. *et al.* A self-limited free-standing sulfide electrolyte thin film for all-solid-state lithium metal batteries. *Adv. Funct. Mater.* **31**, 2101985 (2021).
5. Xu, S., Kwok, C. Y., Zhou, L., Zhang, Z., Kochetkov, I., Nazar, L. F. A high capacity all solid-state Li-sulfur battery enabled by conversion-intercalation hybrid cathode architecture. *Adv. Funct. Mater.* **31**, 2004239 (2021).
6. Yao, X. *et al.* High-performance all-solid-state lithium–sulfur batteries enabled by amorphous sulfur-coated reduced graphene oxide cathodes. *Adv. Energy Mater.* **7**, 1602923 (2017).
7. Ulissi, U., Ito, S., Hosseini, S. M., Varzi, A., Aihara, Y., Passerini, S. High capacity all-solid-state lithium batteries enabled by pyrite-sulfur composites. *Adv. Energy Mater.* **8**, 1801462 (2018).
8. Yamada, T. *et al.* All solid-state lithium–sulfur battery using a glass-type P_2S_5–Li_2S electrolyte: benefits on anode kinetics. *J. Electrochem. Soc.* **162**, A646-A651 (2015).
9. Liu, Z. *et al.* Anomalous high ionic conductivity of nanoporous β-Li_3PS_4. *J. Am. Chem. Soc.* **135**, 975-978 (2013).
10. Phuc, N. H. H., Totani, M., Morikawa, K., Muto, H., Matsuda, A. Preparation of Li_3PS_4 solid electrolyte using ethyl acetate as synthetic medium. *Solid State Ion.* **288**, 240-243 (2016).
11. Wang, H., Hood, Z. D., Xia, Y., Liang, C. Fabrication of ultrathin solid electrolyte membranes of β-Li_3PS_4 nanoflakes by evaporation-induced self-assembly for all-solid-state batteries. *J. Mater. Chem. A* **4**, 8091-8096 (2016).
12. Lim, H-D. *et al.* Designing solution chemistries for the low-temperature synthesis of sulfide-based solid electrolytes. *J. Mater. Chem. A* **6**, 7370-7374 (2018).
13. Calpa, M., Rosero-Navarro, N. C., Miura, A., Tadanaga, K. Preparation of sulfide solid electrolytes in the Li_2S–P_2S_5 system by a liquid phase process. *Inorg. Chem. Front.* **5**, 501-508 (2018).

14. Calpa, M., Rosero-Navarro, N. C., Miura, A., Tadanaga, K. Instantaneous preparation of high lithium-ion conducting sulfide solid electrolyte $Li_7P_3S_{11}$ by a liquid phase process. *RSC Adv.* **7**, 46499-46504 (2017).
15. Ito, S., Nakakita, M., Aihara, Y., Uehara, T., Machida, N. A synthesis of crystalline $Li_7P_3S_{11}$ solid electrolyte from 1,2-dimethoxyethane solvent. *J. Power Sources* **271**, 342-345 (2014).
16. Xu, R. C., Xia, X. H., Yao, Z. J., Wang, X. L., Gu, C. D., Tu, J. P. Preparation of $Li_7P_3S_{11}$ glass-ceramic electrolyte by dissolution-evaporation method for all-solid-state lithium ion batteries. *Electrochim. Acta* **219**, 235-240 (2016).
17. Yao, X. *et al.* High-energy all-solid-state lithium batteries with ultralong cycle life. *Nano Lett.* **16**, 7148-7154 (2016).
18. Rangasamy, E. *et al.* An iodide-based $Li_7P_2S_8I$ superionic conductor. *J. Am. Chem. Soc.* **137**, 1384-1387 (2015).
19. Phuc, N. H. H., Yamamoto, T., Muto, H., Matsuda, A. Fast synthesis of Li_2S–P_2S_5–LiI solid electrolyte precursors. *Inorg. Chem. Front.* **4**, 1660-1664 (2017).
20. Sedlmaier, S. J. *et al.* Li_4PS_4I: A Li^+ Superionic conductor synthesized by a solvent-based soft chemistry approach. *Chem. Mater.* **29**, 1830-1835 (2017).
21. Choi, S., Ann, J., Do, J., Lim, S., Park, C., Shin, D. Application of rod-like Li_6PS_5Cl directly synthesized by a liquid phase process to sheet-type electrodes for all-solid-state lithium batteries. *J. Electrochem. Soc.* **166**, A5193-A5200 (2019).
22. Yubuchi, S., Teragawa, S., Aso, K., Tadanaga, K., Hayashi, A., Tatsumisago, M. Preparation of high lithium-ion conducting Li_6PS_5Cl solid electrolyte from ethanol solution for all-solid-state lithium batteries. *J. Power Sources* **293**, 941-945 (2015).
23. Yubuchi, S., Uematsu, M., Deguchi, M., Hayashi, A., and Tatsumisago, M. Lithium-Ion-conducting argyrodite-type Li_6PS_5X (X = Cl, Br, I) solid electrolytes prepared by a liquid-phase technique using ethanol as a solvent. *ACS Appl. Energy Mater.* **1**, 3622-3629 (2018).
24. Rosero-Navarro, N. C., Miura, A., Tadanaga, K. Composite cathode prepared by argyrodite precursor solution assisted by dispersant agents for bulk-type all-solid-state batteries. *J. Power Sources* **396**, 33-40 (2018).

25. Yubuchi, S., Uematsu, M., Hotehama, C., Sakuda, A., Hayashi, A., and Tatsumisago, M. An argyrodite sulfide-based superionic conductor synthesized by a liquid-phase technique with tetrahydrofuran and ethanol. J. Mater. Chem. A ***7***, 558-566 (2019).

26. Chida, S. *et al.* Liquid-phase synthesis of Li_6PS_5Br using ultrasonication and application to cathode composite electrodes in all-solid-state batteries. *Ceram. Int.* **44**, 742-746 (2018).

27. Zhou, L. *et al.* solvent-engineered design of argyrodite Li_6PS_5X (X = Cl, Br, I) solid electrolytes with high ionic conductivity. *ACS Energy Lett.* **4**, 265-270 (2019).

28. Maniwa, R., Calpa, M., Rosero-Navarro, N. C., Miura, A., Tadanaga K. Synthesis of sulfide solid electrolytes from Li_2S and P_2S_5 in anisole. *J. Mater. Chem. A* **9**, 400-405 (2021).

29. Park, K.H. et al. Solution-processable glass LiI-Li_4SnS_4 superionic conductors for all-solid-state Li-Ion batteries. *Adv. Mater.* **28**, 1874-1883 (2016).

30. Song, Y.B. *et al.* Tailoring solution-processable Li argyrodites $Li_{6+x}P_{1-x}M_xS_5I$ (M = Ge, Sn) and their microstructural evolution revealed by cryo-TEM for all-solid-state batteries. *Nano Lett.* **20**, 4337-4345 (2020).

31. Wang, Y., Liu, Z., Zhu, X., Tang, Y., and Huang, F. Highly lithium-ion conductive thio-LISICON thin film processed by low-temperature solution method. *J. Power Sources* **224**, 225-229 (2013).

32. Sakuda, A., *et al.* Mechanochemically prepared Li_2S–P_2S_5–$LiBH_4$ solid electrolytes with an argyrodite structure. *ACS Omega* **3**, 5453-5458 (2018).

33. Richards, W.D., Miara, L.J., Wang, Y., Kim, J.C., and Ceder, G. Interface stability in solid-state batteries. *Chem. Mater*. **28**, 266-273 (2016).

34. Dewald, G.F. et al. (2019). Experimental assessment of the practical oxidative stability of lithium thiophosphate solid electrolytes. *Chem. Mater.* **31**, 8328–8337 (2016).

35. Muramatsu, H., Hayashi, A., Ohtomo, T., Hama, S., Tatsumisago, M. Structural change of Li_2S–P_2S_5 sulfide solid electrolytes in the atmosphere. *Solid State Ion.* **182**, 116-119 (2011).

36. Shi, T. *et al.* High active material loading in all-solid-state battery electrode via particle size optimization. *Adv. Energy Mater.* **10**, 1902881 (2020).
